# Serum methylation of *GALNT9*, *UPF3A*, *WARS,* and *LDB2* as noninvasive biomarkers for the early detection of colorectal cancer and advanced adenomas

**DOI:** 10.1186/s13148-023-01570-1

**Published:** 2023-10-04

**Authors:** María Gallardo-Gómez, Mar Rodríguez-Girondo, Núria Planell, Sebastian Moran, Luis Bujanda, Ane Etxart, Antoni Castells, Francesc Balaguer, Rodrigo Jover, Manel Esteller, Joaquín Cubiella, David Gómez-Cabrero, Loretta De Chiara

**Affiliations:** 1 CINBIO, Universidade de Vigo, Vigo, Spain; 2 Department of Biochemistry, Genetics and Immunology, CINBIO, Universidade de Vigo, Campus As Lagoas-Marcosende s/n. 36310, Vigo, Spain; 3 Translational Oncology Group, Galicia Sur Health Research Institute (IIS Galicia Sur), SERGAS-UVIGO, Vigo, Spain; 4 Department of Medical Statistics and Bioinformatics, Leiden University Medical Centre, Leiden, The Netherlands; 5 Translational Bioinformatics Unit, Navarrabiomed, Complejo Hospitalario de Navarra (CHN), Universidad Pública de Navarra (UPNA), IdiSNA, Pamplona, Spain; 6 Cancer Epigenetics and Biology Program (PEBC), Bellvitge Biomedical Research Institute (IDIBELL), Avinguda de La Granvia, 199. 08908 L’Hospitalet de Llobregat, Barcelona, Spain; 7 Department of Gastroenterology, Biodonostia Health Research Institute, Centro de Investigación Biomédica en Red de Enfermedades Hepáticas y Digestivas (CIBERehd), Universidad del País Vasco (UPV/EHU), San Sebastián, Spain; 8 Department of Surgery, Hospital Universitario Donostia, San Sebastián, Spain; 9 Gastroenterology Department, Hospital Clínic, IDIBAPS, CIBERehd, University of Barcelona, Barcelona, Spain; 10 Department of Gastroenterology, Hospital General Universitario de Alicante, Alicante, Spain; 11 Josep Carreras Leukaemia Research Institute (IJC), Badalona, Barcelona, Catalonia, Spain; 12 Centro de Investigacion Biomedica en Red Cancer (CIBERONC), Madrid, Spain; 13 Institucio Catalana de Recerca i Estudis Avançats (ICREA), Barcelona, Catalonia, Spain; 14 Physiological Sciences Department, School of Medicine and Health Sciences, University of Barcelona (UB), Barcelona, Catalonia, Spain; 15 Department of Gastroenterology, Complexo Hospitalario Universitario de Ourense, Instituto de Investigación Biomédica Galicia Sur, Centro de Investigación Biomédica en Red de Enfermedades Hepáticas y Digestivas (CIBERehd), Ourense, Spain; 16 Biological & Environmental Sciences & Engineering Division, King Abdullah University of Science & Technology, Thuwal, Kingdom of Saudi Arabia; 17 Mucosal & Salivary Biology Division, King’s College London Dental Institute, London, SE1 9RT UK; 18 Servicio de Medicina Digestiva. ISABIAL. Universidad Miguel Hernández, Hospital General Universitario Dr. Balmis, Alicante, Spain; 19 Cancer Genomics Group, Galicia Sur Health Research Institute (IIS Galicia Sur), SERGAS-UVIGO, Vigo, Spain

**Keywords:** Advanced adenomas, Colorectal cancer, Cancer prevention, Circulating cell-free DNA, DNA methylation, Liquid biopsy, Screening, Serum

## Abstract

**Background:**

Early detection has proven to be the most effective strategy to reduce the incidence and mortality of colorectal cancer (CRC). Nevertheless, most current screening programs suffer from low participation rates. A blood test may improve both the adherence to screening and the selection to colonoscopy. In this study, we conducted a serum-based discovery and validation of cfDNA methylation biomarkers for CRC screening in a multicenter cohort of 433 serum samples including healthy controls, benign pathologies, advanced adenomas (AA), and CRC.

**Results:**

First, we performed an epigenome-wide methylation analysis with the MethylationEPIC array using a sample pooling approach, followed by a robust prioritization of candidate biomarkers for the detection of advanced neoplasia (AN: AA and CRC). Then, candidate biomarkers were validated by pyrosequencing in independent individual cfDNA samples. We report *GALNT9*, *UPF3A*, *WARS*, and *LDB2* as new noninvasive biomarkers for the early detection of AN. The combination of *GALNT9*/*UPF3A* by logistic regression discriminated AN with 78.8% sensitivity and 100% specificity, outperforming the commonly used fecal immunochemical test and the methylated *SEPT9* blood test.

**Conclusions:**

Overall, this study highlights the utility of cfDNA methylation for CRC screening. Our results suggest that the combination methylated *GALNT9*/*UPF3A* has the potential to serve as a highly specific and sensitive blood-based test for screening and early detection of CRC.

**Supplementary Information:**

The online version contains supplementary material available at 10.1186/s13148-023-01570-1.

## Background

Colorectal cancer (CRC) is the third cancer with the highest incidence worldwide and the second leading cause of cancer death in both sexes [1]. Diagnosis at advanced symptomatic stages is responsible for low survival (14% for stage IV) compared to 90% five-year survival for stages I and II [2]. Although the implementation of screening programs is related to the reduction in CRC incidence and mortality [3], the overall participation rate in stool-based screening programs using the fecal immunochemical test (FIT) followed by a confirmatory colonoscopy remains modest (49.5% in Europe and 43.8% worldwide) [4]. In CRC screening settings, FIT reports high specificity (95%) and convenient sensitivity (70–75%) for colorectal tumors [5, 6], but moderate-to-low sensitivity for AA (22–44%) [6–8]. The inconsistent sensitivity of the FDA-approved *SEPT9* blood methylation test for the detection of CRC and AA [9, 10] contraindicates its use for screening.

Since the effectiveness of a screening test relies not only on the test performance but also on its acceptance by the target population, test preference for CRC screening has been evaluated. A survey-based study reported as first choice a blood test over a stool one [11]; similarly, among screening-enrolled individuals who refused colonoscopy, 83% preferred a blood-based test over the 15% that chose a fecal test [12]. Therefore, participation in screening programs could significantly improve by offering a noninvasive blood-based test.

Liquid biopsy has emerged as a noninvasive alternative to traditional procedures for sampling. Blood-based screening is easily available, repeatable, and minimally invasive [13]. Circulating cell-free DNA (cfDNA) can be detected in body fluids and reflects alterations occurring during neoplastic transformation, such as aberrant methylation in colorectal carcinogenesis [14, 15]. This fact, together with the fact that this epigenetic mark is stable during DNA extraction, makes methylation a particularly interesting source of biomarkers for CRC. Indeed, alterations in DNA methylation arise during early stages of tumor progression and heterogeneity of the different pathways to CRC is already detectable in adenomas [16, 17]. The Illumina MethylationEPIC BeadChip array combined with sample pooling represents a particularly suitable strategy for the cost-effective analysis of large sample sets aiming to discover differentially methylated signatures [18]. In this study, following a cfDNA pooling strategy, we aimed to identify noninvasive methylation biomarkers for the early detection of both CRC and premalignant advanced adenomas. Here, we report the discovery and independent validation of serum-based methylation biomarkers that provide a new highly specific and sensitive noninvasive test for the screening and early detection of CRC and advanced adenomas.

## Methods

### Study design

**Fig. 1:**
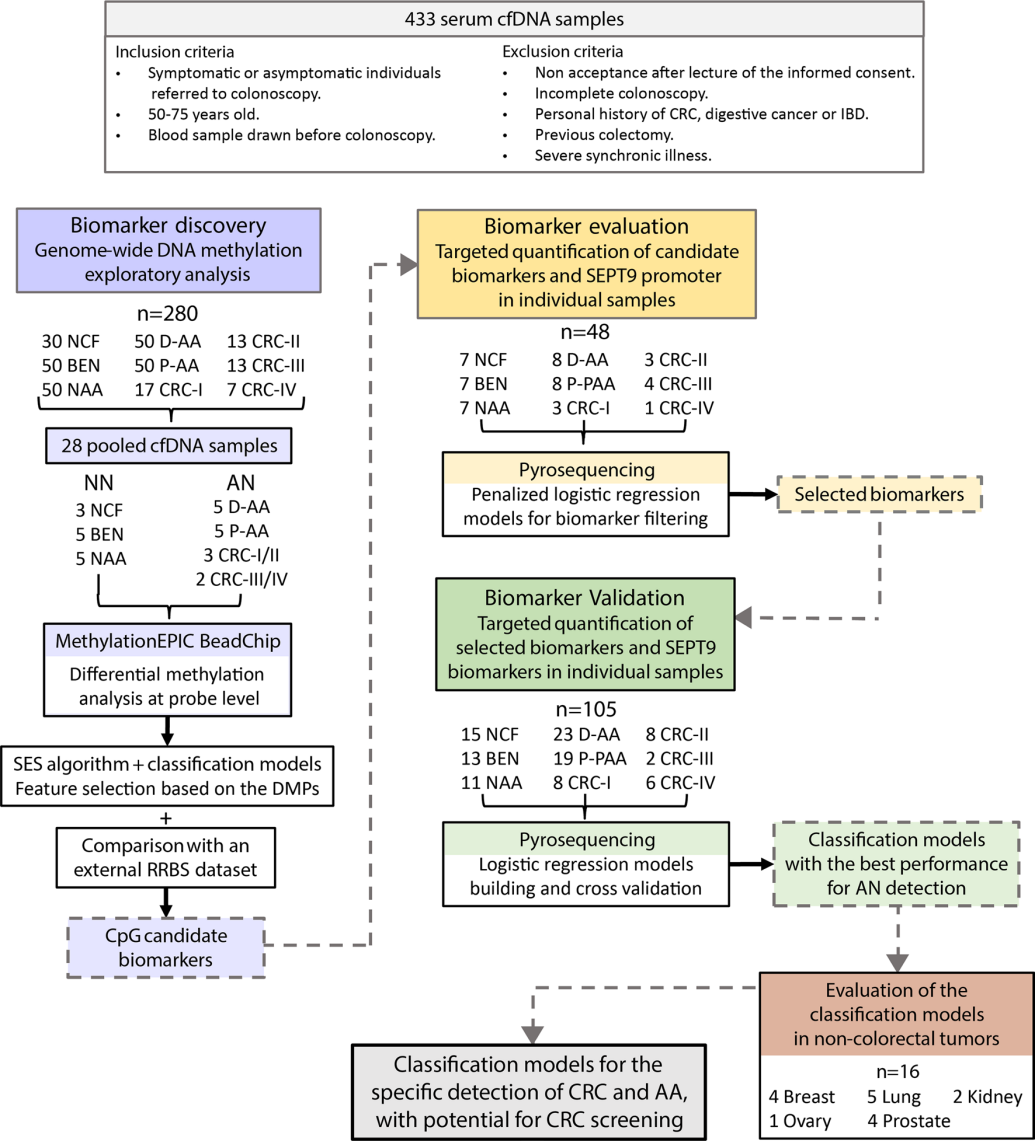
Study workflow. The study was divided into (i) a biomarker discovery phase, (ii) a candidate biomarker evaluation phase, and (iii) a selected biomarker validation. cfDNA: cell-free DNA; CRC: colorectal cancer; IBD: inflammatory bowel disease; NCF: no colorectal findings; BEN: benign pathology; NAA: non-advanced adenomas; D-AA: distal advanced adenomas; P-AA: proximal advanced adenomas; NN: no neoplasia; AN: advanced neoplasia; DMP: differentially methylated position; and RRBS: reduced representation bisulfite sequencing

The study was conducted in three phases: (i) We first performed a high-throughput discovery analysis based on a sample pooling strategy, paired with a statistically robust biomarker prioritization, to identify candidate noninvasive methylation biomarkers for the joint detection of AA and CRC. Next, we designed targeted assays for the quantification of the candidate biomarkers in an independent cohort of patients (individual serum samples). The targeted analysis was divided into (ii) an evaluation of the candidate biomarkers, followed by a feature selection step by penalized logistic regression to obtain specific predictive biomarkers subsets, and (iii) a subsequent validation and final statistical model construction. The final classification models were also evaluated in non-colorectal tumors. An overview of the study design is shown in Fig. 1.

### Patients and samples

Individuals were recruited from the following Spanish Hospitals: Complexo Hospitalario Universitario de Ourense, Hospital Clínic de Barcelona, Hospital Donostia, and Hospital General Universitario de Alicante. A total of 433 symptomatic or asymptomatic individuals between 50 and 75 years old were included. Exclusion criteria comprised a personal history of CRC, digestive cancer or inflammatory bowel disease, a severe synchronic illness, and a previous colectomy. All individuals underwent a colonoscopy, and blood samples were obtained immediately before colonoscopy. Blood samples were coagulated and centrifuged for serum collection. Circulating cell-free DNA (cfDNA) was extracted from 0.5 to 2 mL serum according to availability. Serum samples were stored at − 20 °C until cfDNA extraction.

Individuals were classified according to the most advanced colorectal finding. Advanced adenomas (AA) are defined as adenomas ≥ 1 cm, with villous components or high-grade dysplasia. ‘Advanced colorectal neoplasia’ (AN) was defined as AA or CRC. Individuals with no colorectal findings (NCF), benign pathologies (BEN: hemorrhoids and diverticula), and non-advanced adenomas (NAA) were considered together as ‘no neoplasia’ (NN). Lesions were considered ‘proximal’ when located only proximal to the splenic flexure of the colon and ‘distal’ when found only in the distal colon or in both distal and proximal colon.

We performed a stratified random sampling using colorectal finding and sex as stratifying variables. Strata were matched by age and recruitment hospital. This multicenter cohort was separated into two independent subsets: Discovery cohort (*n* = 280; 140 female and 140 male) and Biomarker validation sample set (*n* = 153; 73 female and 80 male). The latter was randomly split between a Biomarker evaluation cohort (30%, *n* = 48) and a Model validation cohort (70%, *n* = 105) (Table 1).

**Table 1:** Independent cohorts used for the discovery, evaluation, and validation of biomarkers

	Discovery cohort * n* = 280		Biomarker evaluation cohort * n* = 48		Model validation cohort * n* = 105	
	AN	NN	AN	NN	AN	NN
Total (n)	150	130	27	21	66	39
Age median (range)	62.0 (51–72)	62.0 (51–72)	62.5 (50–75)	62.0 (51–75)	63.5 (50–75)	63.0 (50–72)
Sex						
Male	75	65	14	10	36	20
Female	75	65	13	11	30	19
NCF	–	30	-	7	-	15
BEN	–	50	-	7	-	13
Hemorrhoids	–	25	-	3	-	8
Diverticula	–	25	-	4	-	5
NAA	–	50	-	7	-	11
AA	100	–	16	–	42	–
AA histology		–				
TA (size > 10mm)	52	–	11	–	24	–
TVA	36	–	3	–	15	–
VA	12		2	–	3	–
AA dysplasia						
LGD	96	–	13	–	36	–
HGD	4	–	3	–	7	–
AA localization						
Distal	50	–	8	–	23	–
Proximal	50	–	8	–	19	–
CRC	50	–	11	–	24	–
CRC AJCC stage						
Stage I	16	–	3	–	8	–
Stage II	14	–	3	–	8	–
Stage III	14	–	4	–	2	–
Stage IV	6	–	1	–	6	–
CRC localization						
Distal	39	–	9	–	12	–
Proximal	11	–	2	–	12	–

The number of patients, age median and range, sex, and colorectal finding are provided for each sub-cohort. AN: advanced neoplasia; NN: no neoplasia; NCF: no colorectal findings; BEN: benign pathology; NAA: non-advanced adenomas; D-AA: distal advanced adenomas; P-AA: proximal advanced adenomas; TA: tubular adenoma; TVA: tubulovillous adenoma; VA: villous adenoma; LGD: low-grade dysplasia; HGD: high-grade dysplasia; CRC: colorectal cancer; and AJCC: American Joint Committee on Cancer staging system

Methylation microarray data were compared with an external cohort of patients from Hospital Clínic de Barcelona (*n* = 71). Serum and tissue methylation of these patients was quantified by Reduced Representation Bisulfite Sequencing (RRBS), targeting CpG-rich regions of 20 AA and 27 CRC cases, with matched adenoma/tumor, healthy mucosa, and serum samples of each patient, and 24 serum samples from healthy controls.

The specificity of the biomarkers for the detection of CRC and AA was evaluated in an independent cohort of 16 patients with different cancer types (breast, kidney, lung, ovary, and prostate cancer) (Additional file 1: Table S4). Additionally, 8 pairs of matched serum and plasma samples were used to account for differences in the methylation levels between serum and plasma (3 NCF, 2 BEN, and 3 NAA).

### DNA extraction and sample pooling

cfDNA from samples used for biomarker discovery was extracted with a phenol–chloroform protocol [19]. DNA was quantified using Qubit dsDNA HS Assay Kit (Thermo Fisher Scientific, Waltham, MA, USA). For methylation biomarker discovery, we followed a DNA pooling strategy as previously described [18]. Twenty-eight independent cfDNA pooled samples were constructed using equal amounts of cfDNA from 5 men and 5 women from the same pathological group, recruitment hospital- and age-matched. Pools were divided into NN and AN. The NN group comprised 13 cfDNA pooled samples: 3 pools of NCF individuals, 5 pools of BEN, and 5 pools of NAA. On the other hand, the 15 cfDNA pooled samples of AN included 5 pools of proximal AA (P-AA), 5 pools of distal AA (D-AA), 3 pools of CRC stages I-II, and 2 pools of CRC stages III-IV (Additional file 1: Table S2). The cfDNA pools were stored at – 20 °C and were sent to the Cancer Epigenetics and Biology Program facilities at the Bellvitge Biomedical Research Institute (IDIBELL, Barcelona, Spain) for processing and methylation quantification.

The QIAmp Circulating Nucleic Acid Kit (Qiagen, Hilden, Germany) was used for cfDNA extraction from serum and plasma samples in the evaluation and validation phase. Individual cfDNA samples were bisulfite-converted using EZ DNA Methylation-Direct Kit (Zymo Research, Irvine, CA, USA) and stored at – 80 °C.

### Genome-wide DNA methylation measurements

DNA methylation of pooled samples was measured with the Infinium MethylationEPIC BeadChip array (Illumina, San Diego, CA, USA) following the manufacturer’s instructions. A total of 865,859 CpG sites were quantified throughout the genome. Importantly, to minimize the potential impact of batch effects and confounder variability, samples of each pathological group were randomly allocated to the slides. That is, for each beadchip array, we randomly selected one sample from each of the pathological groups, avoiding hybridizing in the same beadchip cfDNA pools from the same pathological group.

### Methylation biomarker discovery

Illumina methylation data were preprocessed and analyzed using the R/Bioconductor environment (see Additional file 1 for details on quality control and preprocessing). To test for differentially methylated positions (DMPs) between AN and NN linear models were fitted for each CpG site across all samples by generalized least squares, and an empirical Bayes method was used to compute the p values. Linear regression assumptions were checked for each model [20].

To select and prioritize the DMPs as candidate biomarkers, we first applied the constraint-based statistically equivalent signature (SES) algorithm for feature selection [21]: Multiple CpG sets with minimal size and maximal predictive power for the binary classification problem NN vs AN were obtained by iteratively comparing logistic regression models through a chi-square test. Then, the different CpG subsets were used to build classification models based on support vector machine, random forest, and logistic regression. Models were cross-validated to select candidate CpG biomarkers with maximal methylation differences and minimum prediction error for NN vs AN classification (see Additional file 1 for details).

### Targeted methylation quantification by bisulfite pyrosequencing

The methylation levels of the candidate biomarkers, together with the v2 promoter region of the SEPT9 gene, were evaluated by bisulfite pyrosequencing in the 153 individual serum samples from the biomarker validation cohort. Bisulfite-converted cfDNA (2 μl) was subjected to PCR amplification using primers flanking the CpG candidate biomarkers. Multiplex reactions including 3–6 candidate markers were performed, followed by nested singleplex PCR reactions using a biotin-labeled primer. Primers and PCR conditions for multiplex and singleplex PCR are provided in Additional file 1 and Additional file 1: Table S3. Pyrosequencing was performed using a PyroMark Q96 ID pyrosequencer (Qiagen, Hilden, Germany). Data acquisition and methylation measurements were conducted at the Biomedical Research Institute of Malaga facilities (IBIMA, Málaga, Spain) using PyroMark Q96 ID software, CpG analysis mode (v1.0.11).

### Biomarker selection and classification model development

Raw and log10-transformed methylation percentages were subjected to multivariate analyses for the development and validation of methylation-based classification models. The validation set of 153 individual samples was randomly divided for the multi-step process of methylation panel development (Table 1).

First, a preliminary evaluation of the methylation levels of the candidate biomarkers was carried out in a 30% sample subset (*n* = 48: 21 NN, 8 D-AA, 8 P-AA, and 11 CRC cases). Penalized logistic regressions, Least Absolute Shrinkage and Selection Operator (LASSO) and Elastic net, were applied to the candidate biomarkers, age, and sex for feature selection, with the *glmnet* package [22]. The minimum mean cross-validation error was used to define the penalty factor. Biomarkers present in the LASSO and Elastic net-derived models were selected for validation. Then, multivariate logistic regressions were fitted in the remaining 70% of samples (*n* = 105: 39 NN, 23 D-AA, 19 P-AA, and 24 CRC cases) to derive models based on the selected biomarkers.

### Statistical analyses

All statistical analyses were performed with the R environment (v3.4.0). In the epigenome-wide methylation analyses, *p* values were adjusted for multiple testing with the Benjamini–Hochberg procedure to control the false discovery rate (FDR). One-sided Fisher’s exact tests were used to assess the significance of the enrichment of the DMPs to functionally annotated elements. To assess the performance of the classification models, receiver operating characteristic (ROC) curves were elaborated, derived by the leave-one-out cross-validation approach, and AUC, sensitivity, specificity, negative and positive predictive values (NPV, PPV) were estimated with their corresponding 95% confidence intervals. The best cutoff values were determined by the Youden index method [23]. Fisher’s exact tests were employed to compare the proportion of distal and proximal lesions detected. Wilcoxon rank-sum test was used to compare the methylation levels between NN and AN in individual serum samples. Nonparametric Wilcoxon signed-rank test was used to compare methylation levels between matched serum and plasma samples.

## Results

### Sample pooling

A total of 28 cfDNA pooled samples (NN group 13 pools and AN 15 pools; cfDNA quantity 62–403 ng; age range from individuals 51–72) were used in the epigenome-wide methylation analysis for biomarker discovery (Additional file 1: Table S2). There was no statistically significant difference in the mean age between pools (ANOVA, *p* value > 0.05). The age range matches the USPSTF guideline recommendation for CRC screening [24].

### Epigenome-wide biomarker discovery

MethylationEPIC BeadChip was used for quantitative DNA methylation profiling in the 28 cfDNA pooled samples. We correctly detected 99.95% of the total array probes. Failed positions and probes not holding the assumptions for linear regression model fitting were discarded. After quality control and preprocessing, a total of 741,310 CpG sites mapped to the human genome assembly GRCh37/hg19 were left for differential methylation testing. No samples were removed due to quality issues.

**Fig. 2:**
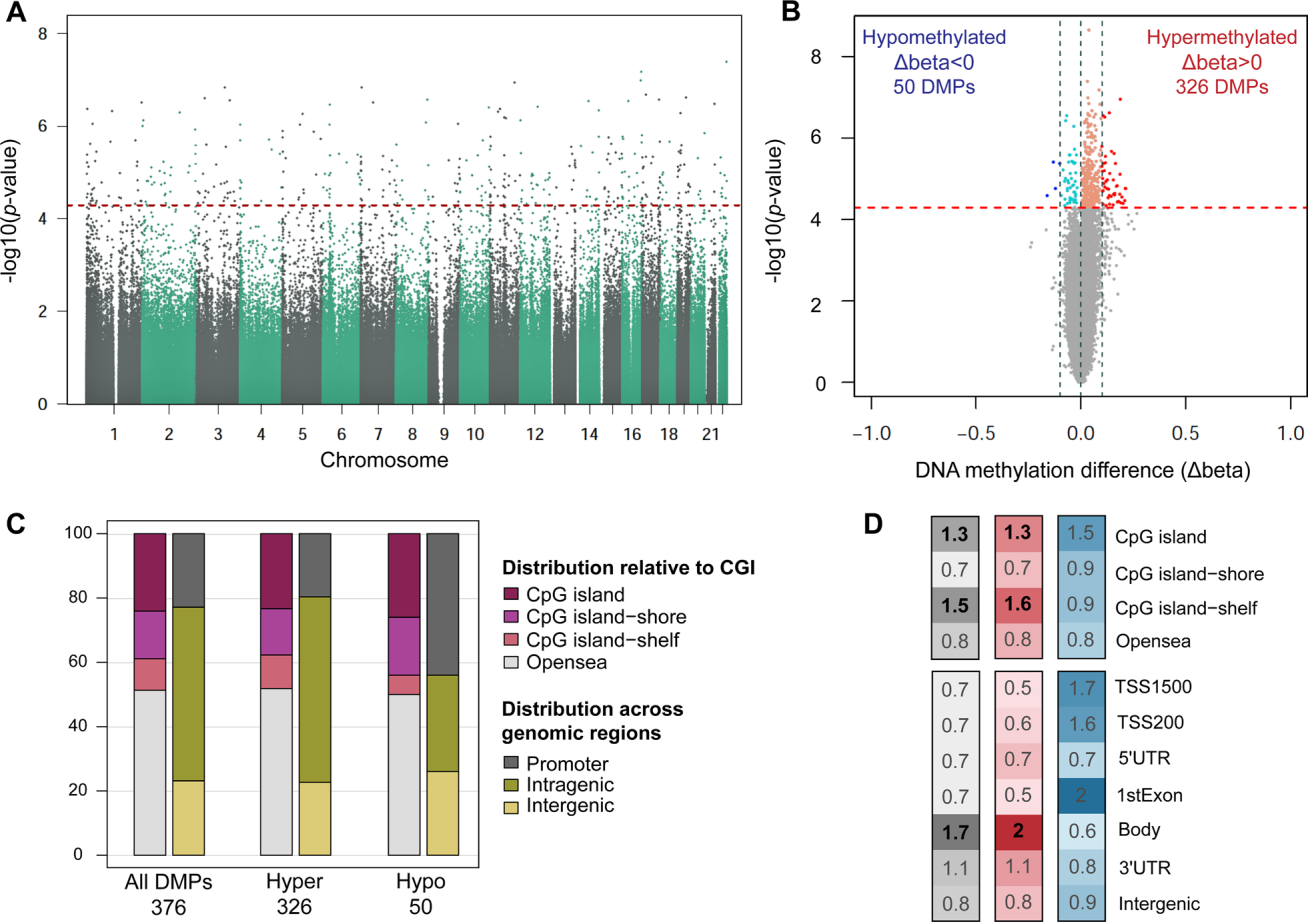
Distribution and annotation of differentially methylated positions (DMPs) between advanced neoplasia and no neoplasia pools. **A** Manhattan plot for differential methylation. The -log_10_(*p* value) for the 741,310 probes analyzed are sorted by chromosome location. Significant DMPs (376) appear above the red dashed line (FDR 10%) **B** Volcano plot showing the -log_10_(*p* value) versus differences in methylation levels (Δbeta: obtained by subtracting the DNA methylation levels (beta-values) of NN from AN). Significant hypermethylated (Δbeta > 0) and hypomethylated (Δbeta < 0) positions appear highlighted in color and above the red dashed line (FDR 10%). **C** Distribution of the DMPs relative to CpG islands and functional genomic locations. **D** Enrichment of DMPs in relation to CpG island annotation and functional genomic regions. The color scale indicates the fold enrichment of all DMPs (gray), hypermethylated (red), and hypomethylated (blue) positions. The bolded numbers indicate annotations that are enriched with respect to the distribution of probes on the MethylationEPIC array (one-sided Fisher’s exact test *p* value < 0.05). Functional characterization of probes according to the Methylation EPIC Manifest: CpG island: region of at least 200bp with a CG content > 50% and an observed-to-expected CpG ratio ≥ 0.6; CpG island-shore: sequences 2 kb flanking the CpG island, CpG island-shelf: sequences 2 kb flanking shore regions, opensea: sequences located outside these regions, promoter regions (5′UTR, TSS200, TSS1500, and first exons), intragenic regions (gene body and 3′UTR), and intergenic regions. TSS200, TSS1500: 200 and 1500 bp upstream the transcription start site, respectively

**Fig. 3:**
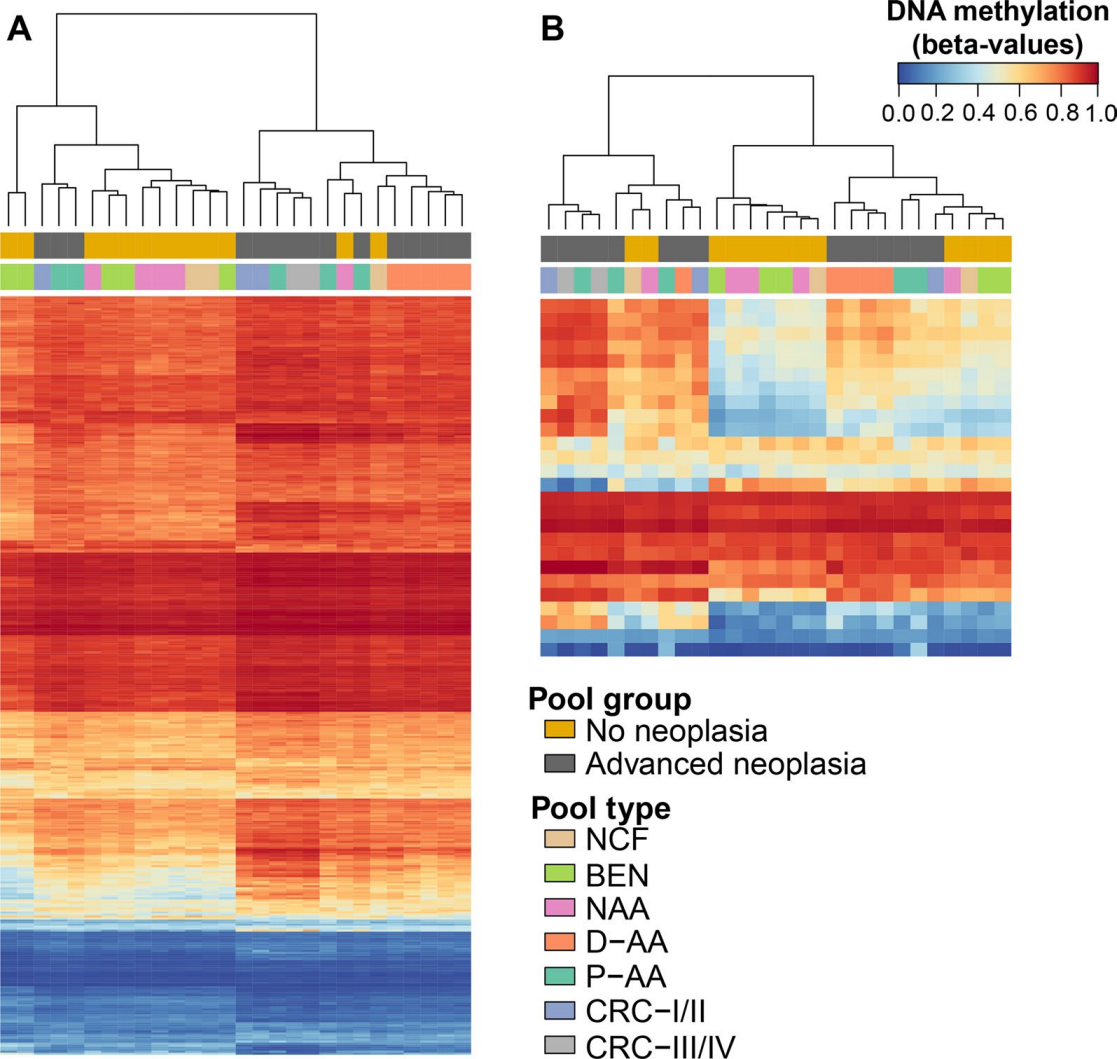
Unsupervised clustering analyses of the 28 cfDNA pooled samples. **A** Hierarchical clustering and heatmap showing the methylation levels across all samples for the 376 DMPs. **B** Hierarchical clustering and heatmap showing the methylation levels across all samples for the 26 candidate biomarkers. Each column represents one pool, while rows correspond to CpG sites. Dendrograms were computed and reordered using Euclidean distance and a complete clustering agglomeration. Methylation levels are expressed as beta-values ranging from 0 (blue, unmethylated) to 1 (red, fully methylated)

Since the purpose of screening programs includes the early detection of preclinical CRC and the detection and removal of AA [25], differential methylation was assessed between NN and AN groups. This analysis revealed 376 differentially methylated positions (10% FDR, BH-adjusted *p* value) (Fig. 2A), annotated to a total of 290 gene regions and 183 CpG islands. Most CpG sites (326 DMPs, 86.7%) were found hypermethylated in AN (Fig. 2B). Concerning the distribution across functional elements, DMPs were mainly located in open sea regions (51.59%) and CpG islands (23.67%) (Fig. 2C). Differentially hypermethylated positions were significantly enriched in CpG islands, shelves, and gene body regions (Fig. 2D). Clustering analyses of all pooled samples based on the methylation values of the 376 DMPs (Fig. 3A) suggest the capacity of this differentially methylated signature to discriminate AN from NN controls.

To identify the most relevant features, a robust strategy of selection was followed. First, the constraint-based SES algorithm was applied to the 376 DMPs to identify 3,256 combinations of CpG pairs whose performances for the NN vs AN classification were statistically equivalent. CpG sites were ranked according to their prediction error (logistic regression, random forest, and SVM models based on CpG pairs) and their absolute methylation differences. From the ranked list, we selected the top 15 CpG sites with greater methylation differences and present in models with minimum classification error. Finally, due to the limited sensitivity of FIT for the detection of AA, we also selected 3 additional CpG sites that presented 0% prediction error for the detection of AA.

In parallel, our data were compared to an external RRBS dataset, comprising serum and tissue samples from patients with CRC, AA, and healthy controls. We selected a subset of 8 CpG sites that reported more than 30% differences in the methylation levels by bisulfite sequencing, showing the same direction (hyper/hypomethylated) in our methylation microarray data.

**Fig. 4:**
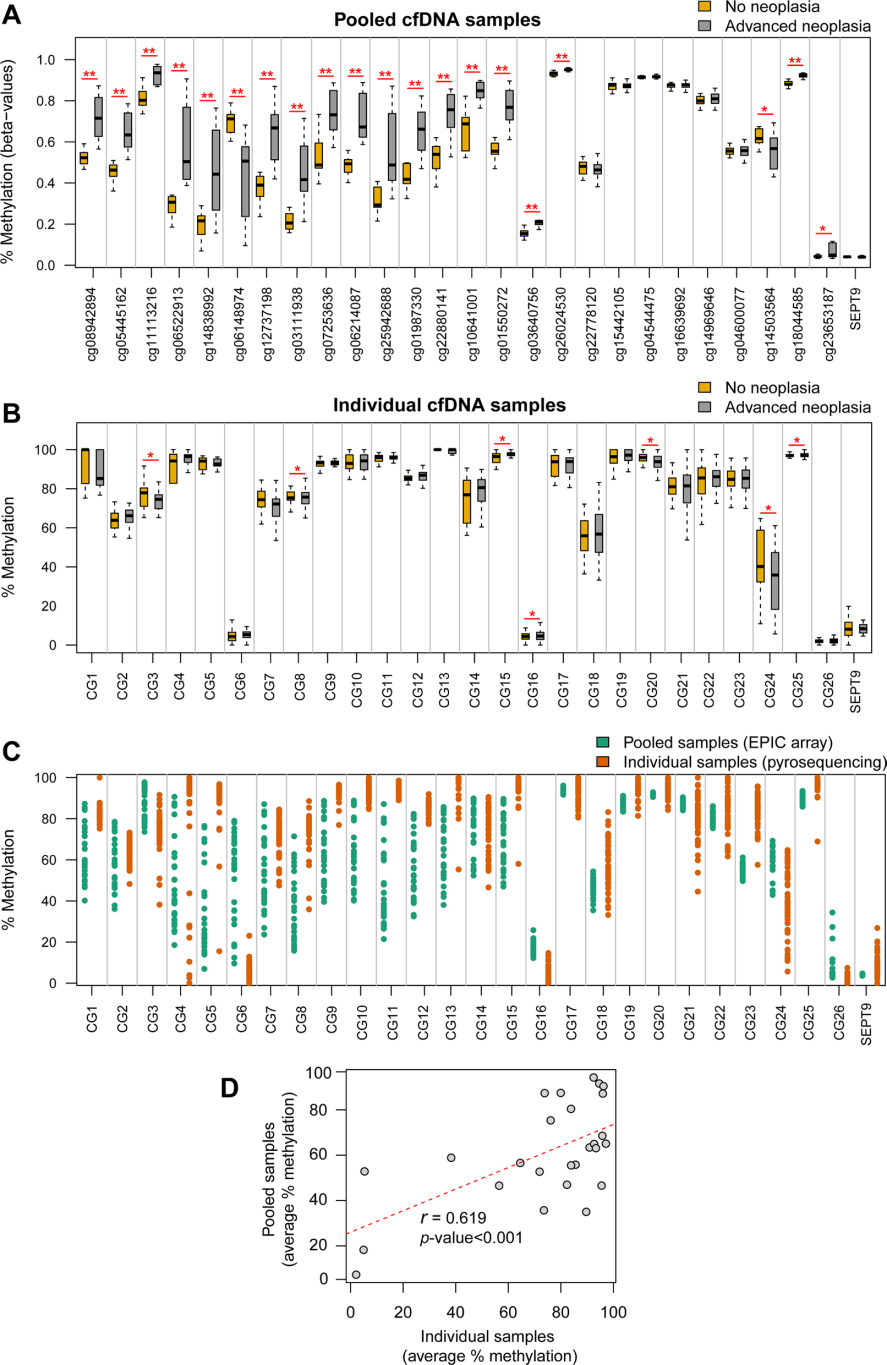
Methylation levels of the 26 candidate biomarkers. **A** Methylation levels of the 26 candidate CpG sites in the 28 pooled cfDNA samples, with the corresponding MethylationEPIC CpG probe ID. Average methylation of the probes targeting the v2 promoter region of the *SEPT9* gene (cg02884239, cg20275528, and cg12783819) is also shown. Methylation is shown as beta-values ranging from 0-unmethylated to 1-fully methylated (**differential methylation *p* value < 0.01; *differential methylation *p* value < 0.05). **B** Methylation levels of the 26 candidate CpG sites and *SEPT9* promoter in the biomarker evaluation cohort (*n* = 48) of individual serum cfDNA samples. Methylation percentage was obtained through bisulfite pyrosequencing (*Wilcoxon rank-sum test *p* value < 0.05). **C** Strip-plot showing the concordance of methylation levels between pooled and individual samples. Each dot represents the methylation level of one sample. **D** Scatterplot shows the positive significant correlation between methylation in pooled and individual cfDNA samples for the 26 candidate CpG sites

Altogether, a total of 26 CpG positions were selected as candidate biomarkers to discriminate colorectal advanced neoplasia (Fig. 3B). Three of 26 markers were hypomethylated, while the rest were found hypermethylated in AN compared to NN (Fig. 4A). Description, regulatory features, and relation to CpG island are available in Additional file 1: Table S4.

### Evaluation of candidate methylation biomarkers and further selection in individual samples

The methylation levels of the 26 candidate biomarkers and the v2 promoter region of the *SEPT9* gene were first quantified in the individual samples from the biomarker evaluation cohort (*n* = 48). Pyrosequenced regions are detailed in Additional file 1: Table S3. The methylation levels of the 26 candidate biomarkers in both pooled (MethylationEPIC-derived) and individual cfDNA samples (bisulfite pyrosequencing) are shown in Figs. 4A and B, respectively. There was a significant positive correlation (Pearson’s r > 0.6, *p* value < 0.001) between cfDNA methylation in pooled and individual serum samples (Fig. 4C, D). The performance (ROC curves, AUC, sensitivity, and specificity) of the 26 biomarkers for the detection of AN in the biomarker evaluation cohort is shown in Additional file 1: Figure S1.

To further reduce the number of amplicons for validation, penalized logistic regressions, LASSO and Elastic net, were fitted to the complete list of 26 candidate biomarkers. Although only CG3, CG8, CG15, CG16, CG20, CG24, and CG25 reported statistically significant methylation differences (Wilcoxon rank-sum rest *p* value < 0.05) between NN and AN (Fig. 4B), variable selection was applied to the whole set of 26 candidate biomarkers since it has been reported that prediction power is not always increased with variables significantly correlated with the outcome [26]. We produced sparse models containing two (CG3-*GALNT9* and CG15-*UPF3A*; AUC: 0.905, 95% CI 0.801–1) and four (CG3-*GALNT9*, CG15-*UPF3A*, CG5-*WARS,* and CG24-*LDB2*; AUC: 0.827, 95% CI 0.651–1) methylation biomarkers, derived by LASSO and Elastic net regularization, respectively. Also, the application of Elastic net to the 26 biomarkers, including age and sex, generated a model containing 20 biomarkers and sex (AUC: 0.820, 95% CI 0.675–0.966). The four biomarkers selected by the sparse models are included in the 20-biomarker signature. Hence, this 20-biomarker set (Table 2) was selected to proceed with the validation phase.

**Table 2:** 20 biomarkers selected for the validation phase

	MethylationEPIC probe ID	Gene symbol	Pyrosequenced region (GRCh37/hg19)	CpG sites analyzed	Relation to CpG island	Regulatory feature
CG2	cg05445162	*GCKR*	chr2:27,730,152–27730225	1	Opensea	Body
CG3	cg11113216	*GALNT9*	chr12:132,847,616–132,847,751	3	Island	Body
CG4	cg06522913	*IDH2*	chr15:90,630,673–90630817	1	Opensea	Body
CG5	cg14838992	*WARS*	chr14:100,814,702–100814791	3	Opensea	Body
CG6	cg06148974		chr4:1,864,164–1,864,242	3	Island	
CG8	cg03111938		chr6:169,289,158–169,289,293	5	Island-shelf	
CG9	cg07253636	*NOSIP*	chr19:50,077,734–50077874	2	Opensea	5'UTR
CG11	cg25942688	*CENPA*	chr2:27,016,702–27016821	1	Opensea	3'UTR
CG12	cg01987330		chr3:142,797,286–142,797,375	1	Opensea	
CG14	cg10641001	*RDX*	chr11:110,104,045–110104122	2	Opensea	Body
CG15	cg01550272	*UPF3A*	chr13:115,050,863–115050975	1	Island-shelf	Body
CG16	cg03640756	*PCDHG* gene cluster	chr5:140,864,546–140864667	11	Island	Body
CG17	cg26024530		chr10:130,281,707–130281864	3	Opensea	
CG19	cg15442105	*ZNF498*	chr7:99,227,410–99227486	4	Opensea	Body
CG20	cg04544475	*RNF43*	chr17:56,435,427–56,435,508	3	Opensea	Body
CG21^†^	cg16639692	lncRNA *LOC648987*	chr5:43,037,639–43037813	1	Island-shore	
CG23^†^	cg04600077	lncRNA *ENSG00000249966*	chr5:1,852,792–1,852,864	1	Island-shore	
CG24^‡^	cg14503564	*LDB2*	chr4:16,723,341–16,723,457	6	Opensea	Body
CG25^§^	cg18044585	*EIF2C2*	chr8:141,619,395–141,619,528	6	Opensea	Body
CG26^‡^	cg23653187	*PNPLA3*	chr22:44,319,222–44,319,299	2	Island-shore	TSS1500

Regulatory features and relation to CpG island of biomarkers annotated according to the Methylation EPIC Manifest: CpG island: region of at least 200 bp with a CG content > 50% and an observed-to-expected CpG ratio ≥ 0.6; Island-shore: sequences 2 kb flanking the CpG island; Island-shelf: sequences 2 kb flanking shore regions; Opensea: sequences located outside these regions; Body: gene body (intragenic region); TSS1500: 1500 bp upstream the transcription start site. †Candidate biomarkers derived from the comparison with the external RRBS dataset; ‡candidate biomarkers derived from the NN vs P-AA classification; §candidate biomarker derived from the NN vs D-AA classification

### Validation of the selected biomarkers and final model construction

**Fig. 5:**
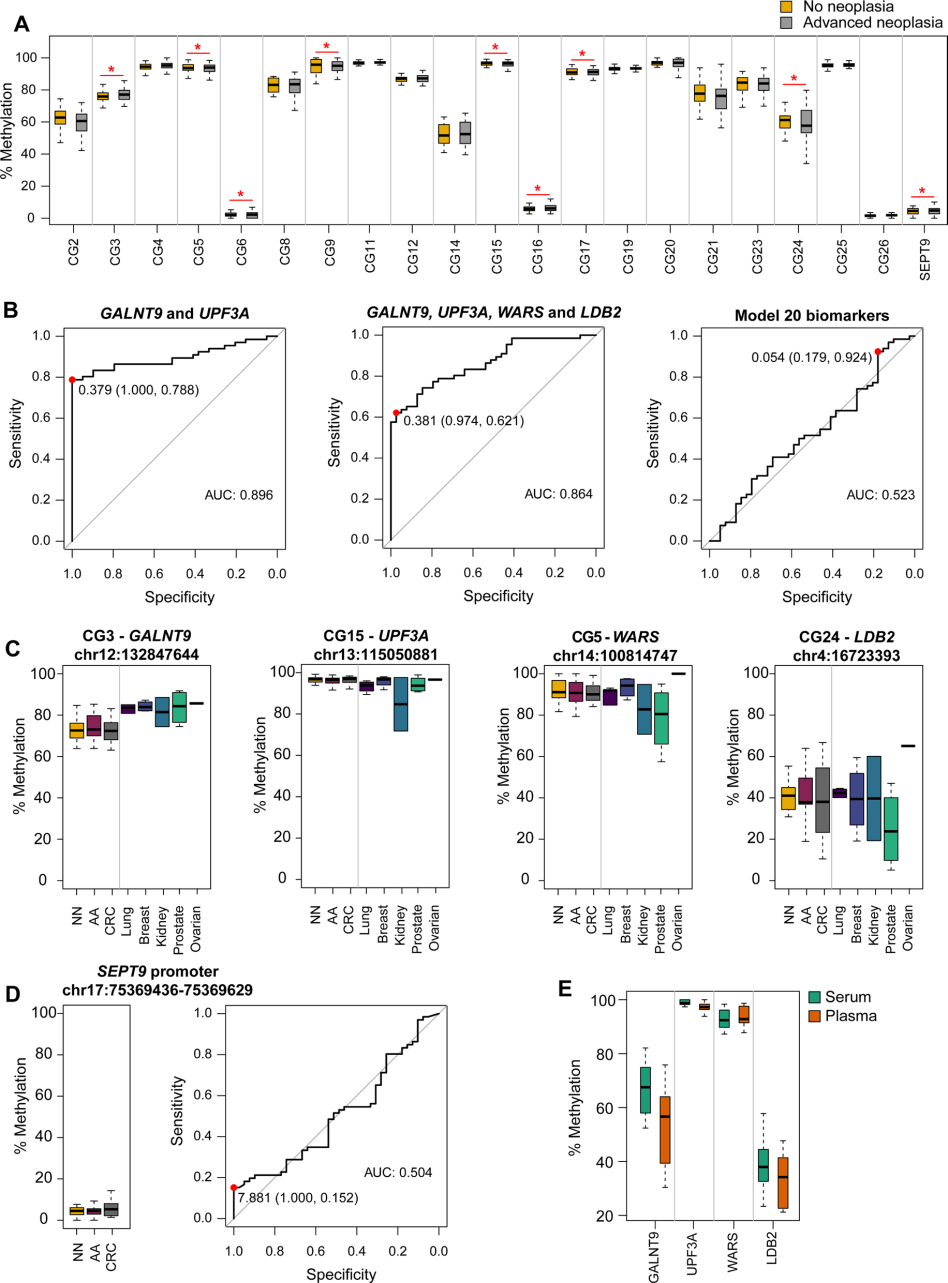
Diagnostic performance of the models and methylation levels in the model validation cohort (*n* = 105). **A** Methylation levels of the final 20 selected biomarkers in the model validation cohort (*Wilcoxon rank-sum test *p* value < 0.05). **B** ROC curve analysis and AUC for the three models evaluated for CRC screening, derived by leave-one-out cross-validation (*GALNT9*: CG3; *UPF3A*: CG15; *WARS*: CG5; and *LDB2*: CG24). The red dots indicate the sensitivity and specificity values for the best cutoffs based on the Youden index method. **C** Serum methylation levels of CG3-*GALNT9*, CG15-*UPF3A*, CG5-*WARS*, and CG24-*LDB2* in the model validation cohort (*n* = 105), and in lung, breast, kidney, prostate, and ovarian cancer cases (*n* = 16). **D** Methylation levels and classification performance (ROC curve) of the *SEPT9* promoter. The red dot indicates the best sensitivity and specificity values (Youden index). **E** Comparison of methylation levels between matched serum and plasma samples. AUC: area under the curve; NN: no neoplasia; AA: advanced adenomas; and CRC: colorectal cancer

The final 20 selected methylation biomarkers were then quantified in the Model validation cohort (*n* = 105) (Fig. 5A). The performance (ROC curves, AUC, sensitivity, and specificity) of the 20 selected biomarkers for the detection of AN is presented in Additional file 1: Figure S2. Multivariate logistic regressions were fitted to the selected biomarker subsets obtained from the three best-performing models from the previous step, to derive three new models containing 2 (CG3-*GALNT9* and CG15-*UPF3A*), 4 (CG3-*GALNT9*, CG15-*UPF3A*, CG5-*WARS*, and CG24-*LDB2*), and 20 biomarkers. Performances of the diagnostic prediction models are summarized in Table 3, while ROC curves are provided in Fig. 5B.

**Table 3:** Performance of the models and *SEPT9* for advanced neoplasia detection in the model validation cohort

	Advanced Neoplasia classification					AA detection %			CRC detection %				
	AUC (95% CI)	Specificity % (95% CI)	Sensitivity % (95% CI)	NPV % (95% CI)	PPV % (95% CI)	D-AA	P-AA	All	Stage I	Stage II	Stage III	Stage IV	All
*GALNT9*, *UPF3A*	0.896 (0.835–0.958)	100 (91–100)	78.8 (67–88)	73.6 (60–85)	100 (93–100)	87	68.4	78.6	87.5	100	100	33.3	79.2
*GALNT9*, *UPF3A*, *WARS*, *LDB2*	0.864 (0.798–0.931)	97.4 (87–100)	62.1 (49–74)	60.3 (47–72)	97.6 (87–100)	78.3	52.6	66.7	37.5	75	100	33.3	54.2
20 methylation biomarkers, sex	0.553 (0.407–0.639)	17.9 (8–34)	92.4 (83–97)	58.3 (28–85)	65.6 (55–75)	91.3	94.7	92.9	87.5	100	100	83.3	91.7
*SEPT9*	0.504 (0.389–0.618)	100 (91–100)	15.1 (8–26)	41.1 (31–52)	100 (69–100)	4.3	10.5	7.1	0	37.5	50	50	29.2

The model validation cohort includes 105 patients. AA and CRC detection rates are also shown. No significant differences were found between the detection of distal versus proximal AA (Fisher’s exact test *p* value > 0.05). ROC curves and performance parameters were derived by the leave-one-out cross-validation approach. AUC: area under the curve; AA: advanced adenoma; CRC: colorectal cancer; NPV: negative predictive value; PPV: positive predictive value; *GALNT9*: CG3; *UPF3A*: CG15; *WARS*: CG5; and *LDB2*: CG24

The model composed of *GALNT9* and *UPF3A* yielded an AUC of 0.896 (95% CI 0.835–0.958), discriminating AN from NN with 78.8% sensitivity and 100% specificity. This model identified 33/42 AA cases (78.6%) and 19/24 CRC patients (79.2%), with notable detection of early-stage CRC (87.5% and 100% for stages I and II, respectively). This 2-biomarker panel detected 87% of distal AA and 68.4% proximal AA. On the other hand, the model containing *GALNT9*, *UPF3A*, *WARS*, and *LDB2* yielded an AUC of 0.864 (95% CI 0.798–0.931), with 62.1% sensitivity and 97.4% specificity for AN, detecting 28/42 AA (66.7%; 78.3% distal and 52.6% proximal), and 13/24 CRC cases (54.2%; 37.5 stage I and 75% stage II). These two models demonstrated no significant differences in the detection of distal AA compared to proximal ones (Fisher’s exact test *p* value > 0.05). Finally, the model containing 20 biomarkers and sex reported the highest sensitivity (92.4%) and the highest detection rate for AA and CRC (92.9% and 91.7%, respectively), with reduced specificity (17.9%). The logistic classification rules of the 4- and 2-biomarker panel are detailed in Additional file 1. CG3-*GALNT9* was hypermethylated in AN, while CG15-*UPF3A*, CG5-*WARS*, and CG24-*LDB2* showed hypomethylation in AN. Differences in methylation levels between NN and AN were statistically significant (Wilcoxon rank-sum test *p* value < 0.05; Fig. 5A). Since plasma samples are most commonly used as a source of cfDNA, methylation of *GALNT9*, *UPF3A*, *WARS*, and *LDB2* was also evaluated in 8 pairs of matched serum and plasma samples. No statistical differences were found between serum and plasma methylation levels (Wilcoxon signed-rank test *p* value > 0.01) (Fig. 5E).

### Analysis and performance of Septin9 methylation

None of the MethylationEPIC probes targeting the v2 promoter of *SEPT9* (cg02884239, cg20275528, cg12783819) reported significance for the detection of AN in the cfDNA pooled samples, showing an average methylation difference of 0.14% between NN and AN (Fig. 4A). We reported hypomethylation of *SEPT9* in AN in both the biomarker evaluation cohort (*n* = 48) (Fig. 4B) and the model validation cohort (*n* = 105) (Fig. 5A). The diagnostic performance of *SEPT9* evaluated in the model validation cohort yielded an AUC of 0.504 (95% CI 0.389–0.618) for AN detection and sensitivity and specificity values of 15.2% and 100%, respectively (Table 3, Fig. 5D). Only 7.1% of AA and 29.2% of CRC were detected.

### Evaluation of the classification models in non-colorectal tumors

To assess the ability of the models to specifically detect AA and CRC, methylation of *GALNT9*, *UPF3A*, *WARS,* and *LDB2* was quantified in serum from patients with lung, breast, kidney, prostate, and ovarian cancer (*n* = 16) (Fig. 5C, Additional file 1: Table S1). The *GALNT9*/*UPF3A* model misclassified as AN 3 out of 16 cancer cases (18.75%: prostate, kidney, and breast cancer). On the other hand, the model composed of *GALNT9*, *UPF3A*, *WARS*, and *LDB2* incorrectly identified 1 out of 16 cancer cases (6.25%: ovarian cancer).

## Discussion

Early detection has proven to be the most effective strategy to reduce both the incidence and mortality of CRC [3, 27]. FIT is the noninvasive screening strategy mostly used for CRC despite having a modest sensitivity for the detection of premalignant AA.[6–8] Also, most existing FIT-based screening programs suffer from low participation rates (43.8% worldwide) [4, 28]. Currently, there is no ideal noninvasive biomarker for the early detection of CRC and AA. To this end, the development of liquid biopsy technology has shown to be a promising approach for CRC screening, diagnosis, follow-up, and treatment guidance [13].

In this study, we first conducted an epigenome-wide analysis with the MethylationEPIC array using a cfDNA pooling approach to discover potential blood-based biomarkers for the joint detection of AA and CRC. The selection process of the candidate biomarkers was conducted by penalized logistic regression. After prioritization of candidate biomarkers and evaluating their methylation levels in individual samples from an independent cohort, we developed and cross-validated three prediction models for the detection of AN (AA and CRC). The first one, the 20 methylation biomarkers with sex, yielded a sensitivity of 92.4% for AN, at 18% specificity. Despite its high sensitivity and highest detection rate for AA (92.9%) and CRC (91.7%), such low specificity is not cost-effective for screening programs. Secondly, the model composed of *GALNT9*, *UPF3A*, *WARS,* and *LDB2* reported 62.1% sensitivity and 97.4% specificity, while the *GALNT9*/*UPF3A* model discriminated AN with 78.8% sensitivity and 100% specificity, showing the best prediction performance for CRC screening. The sensitivities reported for our biomarkers are comparable to that of FIT for CRC detection (70–75%) and higher for AA (22–44%), with increased specificity (97.4–100% and 81–97% for our biomarkers and FIT, respectively) [5–8]. The *GALNT9*/*UPF3A* panel also fulfills the main objective of CRC screening, that is, the detection of preclinical CRC and premalignant AA, as reported suitable detection rate for AA (78.6%) and early CRC stages I and II (87.5% and 100%, respectively). Also, no statistically significant differences were reported between the detection of distal (87%) and proximal (68.4%) AA, in contrast with FIT which performs better for distal lesions [29].

Among the four methylation biomarkers, only *UPF3A* has been previously related to CRC. Located in a CpG island-shelf, we reported the hypomethylation of *UPF3A* in AN. High expression levels of this gene were associated with TNM stage, liver metastasis, and recurrence in CRC [30]. *GALNT9* is also located in a CpG island and showed hypermethylation in AN, which was also reported in brain metastasis from primary breast cancer [31]. On the other hand, *WARS* and *LDB2* show hypomethylation in AN but are located within opensea regions. High expression levels of *WARS* were found in high microsatellite-instable gastrointestinal adenocarcinomas, associated with poor prognosis [32], while a decreased expression of *LDB2* was associated with a more favorable outcome in lung adenocarcinoma patients [33].

Nowadays, the only blood methylation biomarker approved by the FDA for CRC detection is *SEPT9*. Nevertheless, the diagnostic performance of *SEPT9* is variable and inconsistent, with sensitivities ranging from 36–93% for CRC and 22–49% for AA, with 79–99% specificity [9, 10]. In an asymptomatic average-risk cohort, *SEPT9* showed lower performance than FIT (sensitivity: 68% vs. 79%; specificity: 80% vs. 94%, respectively) [10]. In our study, we also evaluated the performance of *SEPT9*. The 3 CpG sites targeting *SEPT9* interrogated in the MethylationEPIC BeadChip were not differentially methylated between NN and AN, and in the final validation cohort, the sensitivity for AN and AA resulted in 15.1% and 7%, respectively, with 100% specificity. Results may not be fully comparable since the commercial test is based on plasma qPCR, while we quantified SEPT9 methylation by pyrosequencing in serum samples.

Several blood-based methylation biomarkers have emerged for CRC detection. Methylated markers such as BCAT1 and IKZF1 [34], C9orf50, KCNQ5, and CLIP4 [35], SFRP2 and SDC2 [36], cg10673833 [37], *APC*, *MGMT*, *RASSF2A*, and *Wif-1* [38], *ALX4*, *BMP3*, *NPTX2*, *RARB*, *SDC2* and *VIM* [39], and *NEUROG1* [40] have reported sensitivities ranging from 66–91% for CRC detection and 5–58% for premalignant AA, with 73–99% specificity.

**Fig. 6:**
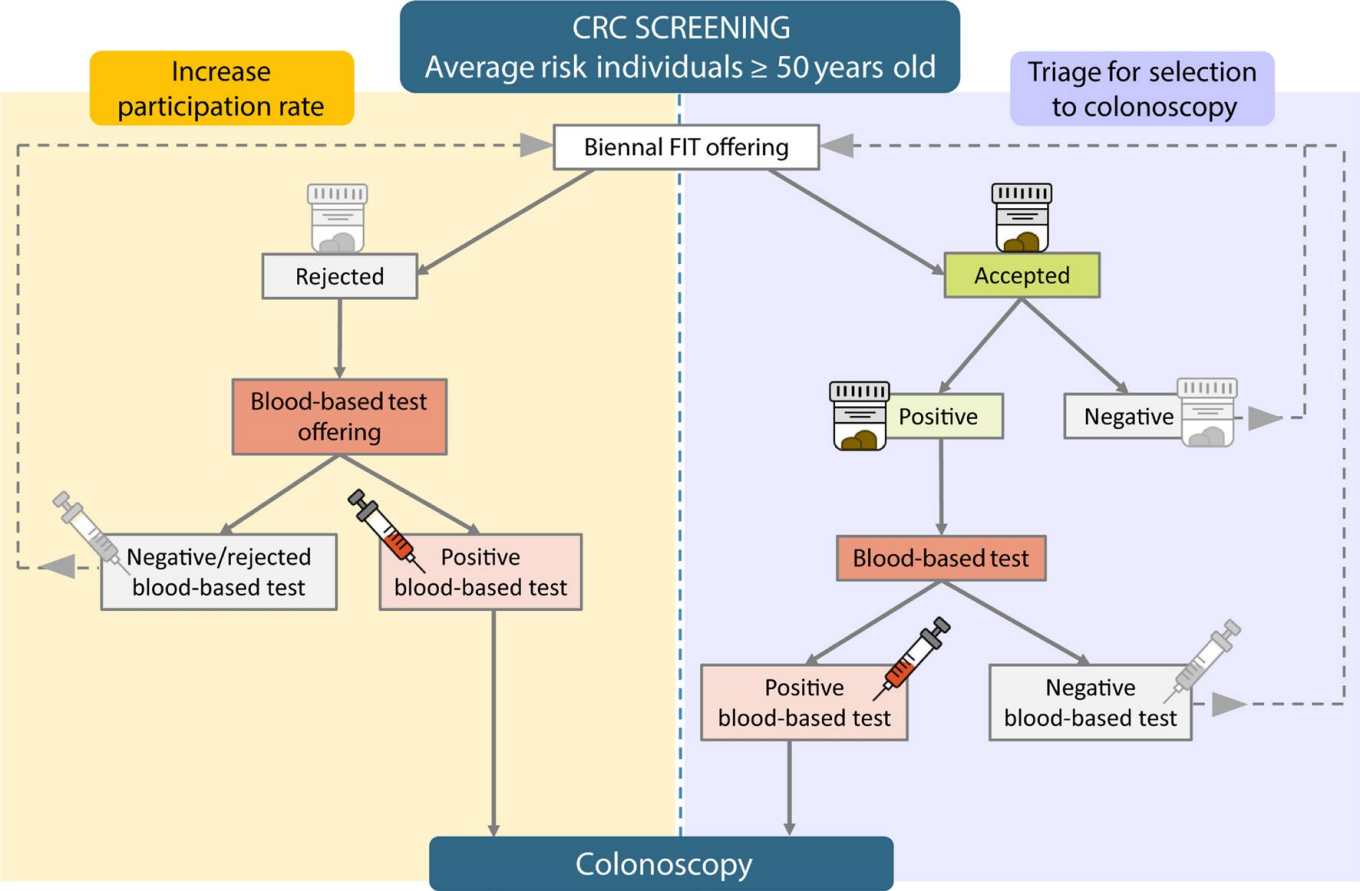
Schematic representation of the potential implementation of a blood-based test in CRC screening. To target different sample preferences and improve participation rates, a blood test could be offered to individuals refusing FIT (left arm). In combination with FIT, the blood test may improve selection for follow-up colonoscopy after a positive FIT (right arm). A FIT test would be offered every two years to individuals rejecting both the FIT and the blood test, and to individuals with a previous negative result in either FIT or blood-based test

In the ongoing validation of CRC screening biomarkers, it is important to consider data on protocol acceptability. A blood-based test has the potential to improve compliance and participation in CRC screening, as reported by a randomized controlled trial [41] and a screening study [12]. To optimize participation in CRC screening, perhaps both a fecal and a blood-based test should be offered to target different preferences. In this way, a blood test could be proposed as a screening option to invitees refusing FIT. Another option for implementing a blood test in screening programs is triaging FIT-positive individuals for improved selection to colonoscopy [42]. Figure 6 shows a schematic representation of the possible implementation of a blood test in CRC screening, both as an alternative to FIT aiming to increase participation rates, and as a triage approach to optimize selection to colonoscopy.

To the best of our knowledge, this is the first study conducting a serum-based discovery and validation of cfDNA methylation biomarkers for CRC screening. Our study design, targeting the final sample format (serum), enhances the possibility to discover and translate robust noninvasive biomarkers. Additionally, our results underline the feasibility of cfDNA pooled samples as an affordable approach for biomarker discovery, increasing the DNA input when small amounts are available [18, 43, 44]. Our multicenter cohort includes colonoscopy-diagnosed individuals with CRC, AA, healthy controls, and also benign pathologies (non-advanced adenomas, hemorrhoids, and diverticula) typically found during screening programs that influence test specificity. The ability of our methylation biomarkers to specifically detect CRC was also confirmed.

Nevertheless, our study has some limitations. Firstly, CRC cases were mostly diagnosed as having symptoms, and secondly, the proportions of CRC cases, tumor stages, and the rest of the pathologies are not fully representative of a screening population. The proposed methylation models should be validated in a large prospective average-risk screening setting.

## Conclusion

We have discovered and reported *GALNT9*, *UPF3A*, *WARS*, and *LDB2* as new noninvasive biomarkers for the early detection of CRC and AA, regardless of the location of the lesion. We propose that the combination of methylated *GALNT9*/*UPF3A* is the most promising to serve as a highly specific and sensitive blood-based test for screening and detection of CRC at an early and curable stage, even at the premalignant lesion phase.

### Supplementary Information

**Additional file 1**. Details about the bioinformatics preprocessing of methylation microarray data for biomarker discovery. Details about biomarker discovery analysis and robust biomarker prioritization. PCR conditions and primers for biomarker evaluation and validation in individual serum samples. Decision rules derived from the final models for de detection of colorectal advanced neoplasia. Table S1. Epidemiological and clinical data of patients with other tumors (*n* = 16). Table S2. Description of cfDNA pooled samples. Table S3. Primers, PCR conditions, and amplicon details for biomarker evaluation by pyrosequencing. Table S4. Description of the CpG candidate biomarkers obtained after the epigenome-wide methylation analysis. Fig. S1. ROC curve analysis for the 26 candidate biomarkers and *SEPT9* for NN versus AN classification in the biomarker evaluation cohort (*n* = 48). Fig. S2. ROC curve analysis for the 20 selected biomarkers and *SEPT9* for NN versus AN classification in the model validation cohort (*n* = 105).

## Data Availability

The Infinium MethylationEPIC data from all the pooled samples generated and analyzed during this study have been deposited in the NCBI Gene Expression Omnibus (GEO) (www.ncbi.nlm.nih.gov/geo) and are accessible through GEO Series accession number GSE186381. [https://www.ncbi.nlm.nih.gov/geo/query/acc.cgi?acc=GSE186381].

## Abbreviations

AA: Advanced adenoma
AN: Advanced neoplasia
AUC: Area under the curve
BEN: Benign pathologies Hemorrhoids and diverticula
cfDNA: Circulating cell-free DNA
CRC: Colorectal cancer
D-AA: Individuals with advanced adenomas of both distal and proximal locations
DMP: Differentially methylated position
FDR: False discovery rate
FIT: Fecal immunochemical test
NCF: No colorectal findings
NN: No neoplasia
NPV: Negative predictive value
PPV: Positive predictive value
ROC: Receiver operating characteristic
