# Supplementary material for: Serum methylation of GALNT9, UPF3A, WARS, and LDB2 as noninvasive biomarkers for the early detection of colorectal cancer and advanced adenomas
Source: Clin Epigenetics. 2023 Oct 4;15:157. doi: 10.1186/s13148-023-01570-1 (PMC10552320; doi:10.1186/s13148-023-01570-1)
Supplement: Supplementary file 1 — Additional file 1. Details about the bioinformatics preprocessing of methylation microarray data for biomarker discovery. Details about biomarker discovery analysis and robust biomarker prioritization. PCR conditions and primers for biomarker evaluation and validation in individual serum samples. Decision rules derived from the final models for de detection of colorectal advanced neoplasia. Table S1. Epidemiological and clinical data of patients with other tumors (n = 16). Table S2. Description of cfDNA pooled samples. Table S3. Primers, PCR conditions, and amplicon details for biomarker evaluation by pyrosequencing. Table S4. Description of the CpG candidate biomarkers obtained after the epigenome-wide methylation analysis. Fig. S1. ROC curve analysis for the 26 candidate biomarkers and SEPT9 for NN versus AN classification in the biomarker evaluation cohort (n = 48). Fig. S2. ROC curve analysis for the 20 selected biomarkers and SEPT9 for NN versus AN classification in the model validation cohort (n = 105). [file 13148_2023_1570_MOESM1_ESM.docx]

**SUPPLEMENTARY MATERIAL**

Details about the bioinformatics preprocessing of methylation microarray data for biomarker discovery. Details about biomarker discovery analysis and robust biomarker prioritization. PCR conditions and primers for biomarker evaluation and validation in individual serum samples. Decision rules derived from the final models for de detection of colorectal advanced neoplasia.

**Supplementary Table 1.** Epidemiological and clinical data of patients with other tumors (n=16). **Supplementary Table 2.** Description of cfDNA pooled samples. **Supplementary Table 3.** Primers, PCR conditions, and amplicon details for biomarker evaluation by pyrosequencing. **Supplementary Table 4.** Description of the CpG candidate biomarkers obtained after the epigenome-wide methylation analysis.

**Supplementary Figure 1**. ROC curve analysis for the 26 candidate biomarkers and *SEPT9* for NN versus AN classification in the biomarker evaluation cohort (n=48). **Supplementary Figure 2.** ROC curve analysis for the 20 selected biomarkers and *SEPT9* for NN versus AN classification in the model validation cohort (n=105).

**Table of content**

| Bioinformatics Preprocessing of Methylation Microarray Data for Biomarker Discovery | p. 2 |
| --- | --- |
| Pcr Conditions and Primers for Biomarker Evaluation | p. 2 |
| Final Models for the Detection of Colorectal Advanced Neoplasia | p. 3 |
| Supplementary Tables | pp. 4-9 |
| Supplementary Figures | pp. 10-13 |
| References | p. 14 |

**SUPPLEMENTARY INFORMATION**

**BIOINFORMATICS PREPROCESSING OF METHYLATION MICROARRAY DATA FOR BIOMARKER DISCOVERY**

Illumina methylation data were preprocessed and analyzed using the R environment (versions 3.3.3 and 3.4.0)^1^ with R and Bioconductor^2^ packages.

**Quality control.** First, data quality control was assessed based on the internal control probes present on the array. Technical variation, outliers, and potential sources of covariates were explored with multi-dimensional scaling plots and principal component analysis. The distribution of methylation levels was checked across all samples.

**Probe filtering.** Detection p values were computed with the *minfi* package,^3^ and mean detection p values were examined across all samples to identify any failed sample. Probe filtering was performed with the *wateRmelon* package.^4^ Probes with a detection p value > 0.01 in at least one sample, probes with a bead count < 3 in at least 1% of samples, and probes that violated any assumption for linear regression model fitting (linearity, homoscedasticity, uncorrelatedness, and normality of the standardized residuals) were discarded. We filtered out probes containing single nucleotide polymorphisms and probes targeting X and Y chromosomes. Previously reported cross-reactive probes^5^ were also removed. No sample was discarded due to quality issues. Probes were annotated to CpG island, genomic regions, and RefSeq genes according to the MethylationEPIC Manifest (R package version 0.6.0).^6^

**Normalization and batch effect correction.** The dataset was then normalized using the single-sample out-of-band normalization method (ssNoob)^7,8^ implemented in the *minfi* package. This approach for background and dye bias correction does not rely on biological assumptions, as it is based on the non-specific fluorescence of Infinium I probes. Before differential methylation analysis, data were adjusted for the BeadChip batch effect using the ComBat method^9^ implemented in the *sva* package.^10^

**Differential methylation analyses**: Methylation levels were expressed as beta-values for visualization and intuitive interpretation of the results. Methylation expressed as M-values (*logit* transformation of the beta-values) were used for differential methylation analysis and biomarker selection.^11^ To test for differentially methylated positions (DMPs) between AN and NN we used the *limma* package:^12^ linear models were fitted for each CpG site across all samples by generalized least squares, and an empirical Bayes method was used to compute the p-values. Linear regression assumptions were checked for each model^13^ using the *gvlma* package.^14^

To select and prioritize the DMPs as candidate biomarkers we first applied the constraint-based Statistically Equivalent Signature (SES) algorithm for feature selection, contained in the *MXM* package:^15^ multiple CpG sets with minimal size and maximal predictive power for the binary classification problem NN vs AN were obtained by iteratively comparing logistic regression models through a chi-square test. Secondly, we used two strategies for filtering:

1. The different CpG subsets were used to build classification models based on support vector machine (*e1071* package),^16^ random forest (*randomForest* package),^17^ and logistic regression. Models were cross-validated to select candidate CpG biomarkers with minimum prediction error for NN vs AN classification. CpG sites present in models with more than 20% prediction error were discarded.
2. Then remaining CpG sites were ranked according to the mean difference in the methylation levels between NN and AN. The top CpG sites from this ranked list were selected for targeted evaluation.

**PCR CONDITIONS AND PRIMERS FOR BIOMARKER EVALUATION**

PCR and sequencing primers were designed with the PyroMark Assay Design software (version 2.0.1.15, Qiagen, Hilden, Germany).

ABM 2x PCR HotStart Mastermix (Applied Biological Materials, Richmond, Canada) was used for PCR amplification with the following cycling conditions:

- Multiplex PCR: 94°C 10 min, 32 cycles of (94°C 30 s, 58°C 30 s, 72°C 30 s), 72°C 5 min, cool down to 4°C. Ordinary primers were used at a final concentration of 0.6 µM
- Singleplex PCR: 94°C 10 min, 20/25 cycles of (94°C 30 s, 58/60°C 30 s, 72°C 30 s), 72°C 5 min, cool down to 4°C. Primers were used at a final concentration of 0.5 µM. A biotin-labelled primer, either forward or reverse (see Supplementary table 3) was used.

Fully methylated and unmethylated controls were included in each PCR amplification batch and pyrosequencing run to check bisulfite conversion, performance of the assay, and to account for batch‐to‐batch variation. For the fully methylated control DNA from a blood donor was treated with CpG methyltransferase (M.SssI; New England Biolabs, Ipswich, MA, USA), while for the unmethylated control whole genome amplification was performed to eliminate methylation marks (illustra GenomiPhi V2 DNA Amplification Kit, GE Healthcare; Chicago, IL, USA).

**FINAL MODELS FOR THE DETECTION OF COLORECTAL ADVANCED NEOPLASIA**

The cut-off value of p for AN classification was determined by the Youden Index method. The final classification models where *p* is the predicted probability of being classified as advanced neoplasia are given by:

*GALNT9/UPF3A* model (AN if p>0.379)

$$logit(p) = -6.223 - 0.0218log(GALNT9 + 1) +1.270log(UPF3A + 1)$$

*GALNT9*, *UPF3A*, *WARS* and *LDB2* model (AN if p>0.381)

$$logit(p) = -2.332 - 0.006GALNT9 +0.020UPF3A +0.002WARS +0.003LDB2$$

The values of CG3-*GALNT9*, CG15-*UPF3A*, CG5-*WARS* and CG24-*LDB2* correspond to the methylation levels of the CpG sites chr12:132847644, chr13:115050881, chr14:100814747 and chr4:16723393, respectively (GRCh37/hg19 coordinates).

**Supplementary Table 1. Epidemiological and clinical data of patients with other tumors (n=16).**

| **Age median (range)** | 61 (35-79) |
| --- | --- |
|  |  |
| **Male** | 8 |
| **Female** | 8 |
|  |  |
| **Breast cancer** | **4** |
| Luminal A, Luminal B | 1 |
| Luminal B, HER2-negative | 3 |
|  |  |
| **Kidney cancer** | **2** |
| Papillary transitional cell carcinoma | 2 |
|  |  |
| **Lung cancer** | **5** |
| Adenocarcinoma | 2 |
| Squamous cell carcinoma | 2 |
| Small cell neuroendocrine carcinoma | 1 |
|  |  |
| **Prostate cancer** | **4** |
| Adenocarcinoma | 3 |
| Acinar adenocarcinoma | 1 |
|  |  |
| **Ovarian cancer** | **1** |
| High grade serous carcinoma | 1 |

**Supplementary table 2. Description of cfDNA pooled samples.**

| **Pool type** | **Age median (range)** | **Total amount DNA (ng)** | **Pathology description** |
| --- | --- | --- | --- |
| NCF | 63 (52-71) | 160.0 | Each pool contained 10 individuals with no colorectal findings |
|  | 62 (53-71) | 179.2 |  |
|  | 60.5 (54-71) | 190.4 |  |
| BEN | 62 (53-71) | 142.6 | Each pool contained 5 individuals with hemorrhoids and 5 individuals with diverticula. |
|  | 61 (51-70) | 164.8 |  |
|  | 62.5 (52-72) | 210.2 |  |
|  | 61.5 (52-70) | 206.1 |  |
|  | 62.5 (52-72) | 403.2 |  |
| NAA | 62 (52-72) | 225.6 | Each pool contained 10 individuals with colorectal tubular adenomas without high-grade dysplasia and less than 10 mm in size. |
|  | 61 (52-71) | 176.0 |  |
|  | 61.5 (52-72) | 195.2 |  |
|  | 62 (51-72) | 214.4 |  |
|  | 61.5 (51-72) | 169.6 |  |
| D-AA | 61.5 (53-72) | 156.6 | Each pool contained 10 individuals with adenomas greater than 10 mm in size, and/or with villous histological component, and/or high-grade dysplasia. The most severe lesion was located in the distal colon, but other adenomas may be present also in the proximal colon. |
|  | 62 (51-71) | 184.0 |  |
|  | 62 (52-70) | 203.2 |  |
|  | 62 (54-70) | 336.0 |  |
|  | 63 (54-69) | 225.6 |  |
| P-AA | 60 (52-72) | 332.8 | Each pool contained 10 individuals with adenomas greater than 10 mm in size, and/or with villous histological component, and/or high-grade dysplasia. All advanced adenomas were of proximal location. |
|  | 61.5 (52-69) | 256.0 |  |
|  | 62.5 (53-72) | 158.1 |  |
|  | 61 (56-70) | 128.2 |  |
|  | 61 (55-71) | 61.9 |  |
| CRC I/II | 60.5 (53-71) | 182.4 | 6 CRC stage I and 4 CRC stage II |
|  | 62 (51-71) | 185.6 | 5 CRC stage I and 5 CRC stage II |
|  | 62 (51-70) | 169.6 | 6 CRC stage I and 4 CRC stage II |
| CRC III/IV | 62.5 (53-72) | 147.0 | 7 CRC stage III and 3 CRC stage IV |
|  | 62.5 (52-71) | 157.5 | 6 CRC stage III and 4 CRC stage IV |

Pools were constructed with equal amounts of cfDNA from 5 men and 5 women from the same pathological group, recruitment hospital- and age-matched. NCF: no colorectal findings; BEN: benign pathology; NAA: non-advanced adenomas; D-AA: distal advanced adenomas; P-AA: proximal advanced adenomas; CRC: colorectal cancer.

**Supplementary table 3. Primers, PCR conditions and amplicon details for biomarker evaluation by pyrosequencing. Biomarkers included in the same multiplex PCR reaction are grouped together. Biotin-labelled primers are highlighted with italics.**

| Biomarkers | Primers | Singleplex PCR temperature (°C) | Singleplex PCR cycles | Singleplex amplicon length (bp) | Pyrosequenced region (GRCh37/hg19) | Pyrosequenced region  length (bp) | Amount of CpG sites analysed |
| --- | --- | --- | --- | --- | --- | --- | --- |
| CG2 | PCR→ GTGATATGTTTAATTAGAAGGTTGAGTTTA  PCR← *CACACTAATAATCTCCCCAACT*  Sequencing→ AATTAGAAGGTTGAGTTTATTAA | 60 | 25 | 85 | chr2:27730152-27730225 | 74 | 1 |
| CG3 | PCR→ TTTAAAAATTAAGTAGAGGGGAGAGTAGGT  PCR← *ACCCCACATAACCACTACTACC*  Sequencing→ AGGGGAGAGTAGGTG | 60 | 25 | 152 | chr12:132847616-132847751 | 136 | 3 |
| CG5 | PCR→ GGGAGAAGTATAATGTTGGGAGGTTTGTAA  PCR← *TCTCCAATACCCCCCAAAAC*  Sequencing→ TTTAGGTTGAGTAGAGGTA | 58 | 25 | 145 | chr14:100814702-100814791 | 90 | 3 |
| CG11 | PCR→ GGGGATTTTTTAGAGTTATGATTAGAT  PCR← *AATCATACAATCTTCTCCTTCTCA*  Sequencing→ GAGTTATGATTAGATTTAATGGA | 60 | 20 | 132 | chr2:27016702-27016821 | 120 | 1 |
| CG21 | PCR→ GTTTTGGGTTTTAGTAAGTTTTATAGAAGT  PCR← *ACTAACCCTCAACTTTATACTATCT*  Sequencing→ GGTTTTAGTAAGTTTTATAGAAGTA | 58 | 20 | 181 | chr5:43037639-43037813 | 175 | 1 |
| SEPT9 | PCR→ *AGGGGGTTTAGGGGTTTTT*  PCR← CCAACCCAACACCCACCT  Sequencing← AAATCCCAAATAATCCCATCC | 58 | 25 | 215 | chr17:75369436-75369629 | 194 | 5 |

**Supplementary table 3 (continuation).**

| Biomarkers | Primers | Singleplex PCR temperature (°C) | Singleplex PCR ycles | Singleplex amplicon length (bp) | Pyrosequenced region (GRCh37/hg19) | Pyrosequenced region  length (bp) | Amount of CpG sites analyzed |
| --- | --- | --- | --- | --- | --- | --- | --- |
| CG8 | PCR→ *GTGTGTTGATTGTGGATAGGT*  PCR← ACAACCATAAAATTCTACTAAATCTAAAC  Sequencing← AATTCTACTAAATCTAAACAATAAT | 58 | 20 | 146 | chr6:169289158-169289293 | 136 | 5 |
| CG9 | PCR→ GGGTTTGGATAGTTATAGGATGT  PCR← *TCCAACCTCAAAAACTAAAAAAAATAAATC*  Sequencing→ ATTGGAAAATATATAGTTGTAGT | 58 | 20 | 175 | chr19:50077734-50077874 | 141 | 2 |
| CG12 | PCR→ TTGTGATTGGTGGTTGTAGGT  PCR← *AACTTCCCTACCCTATATTAAAACCACTA*  Sequencing →GTGGTGAGAAGAAAATAATT | 58 | 25 | 109 | chr3:142797286-142797375 | 90 | 1 |
| CG13 | PCR→ AGATAGGGTTGTTTAGTTTTAATGATAATA  PCR← *ATCTCAAATCTACCCCTCTCAAAAATACAA*  Sequencing→ ATTATTGGTGTTTATTAGTTGA | 58 | 20 | 223 | chr1:28160760-28160884 | 125 | 2 |
| CG15 | PCR→ AGGGAGTATGTTATTTGTTATTGAATGA  PCR← *AATATTTTTATACCAACCTCCACTATC*  Sequencing→ TAGGTTTTGTGGGTG | 60 | 20 | 156 | chr13:115050863-115050975 | 113 | 1 |
| CG1 | PCR→ GGTTTTTGTAAATAGTTGTATTGAAGTAG  PCR← *AACTACTAAATAACACCAACAACATC*  Sequencing→ AAATAGGTATAAAGAAGATTGT | 60 | 20 | 129 | chr15:83563756-83563833 | 78 | 1 |
| CG4 | PCR→ GGGAAGGGAAGAAAGGTTATAGAGTAT  PCR← *CCCCCTACAATCCATCTCAAATTTTAC*  Sequencing→ GTTAAAGATTTTTTGGAAGATG | 60 | 20 | 190 | chr15:90630673-90630817 | 145 | 2 |
| CG18 | PCR→ ATTTGGAGGTTTTGTGTTTGT  PCR← *CTAACCCCCCTAAAACATCAAATAACAATC*  Sequencing→ TTTGTTGGTAGGGGTA | 60 | 25 | 104 | chr22:45094510-45094597 | 88 | 2 |
| CG20 | PCR→ TTGGGTTAGGTTTTTTGTTATGTTATT  PCR← *AATTTCCAACCATATCCACTACC*  Sequencing→ GTTAGGTTTTTTGTTATGTTATTG | 60 | 25 | 86 | chr17:56435427-56435508 | 82 | 3 |
| CG25 | PCR→ GGGGAATTTAGGATGGGTATTATAT  PCR← *CTCAAAATTACCAACTATTTAAACCATACA*  Sequencing→ AATTTAGGATGGGTATTATATT | 60 | 20 | 138 | chr8:141619395-141619528 | 134 | 6 |

**Supplementary table 3 (continuation).**

| Biomarkers | Primers | Singleplex PCR temperature (°C) | Singleplex PCR cycles | Singleplex amplicon length (bp) | Pyrosequenced region (GRCh37/hg19) | Pyrosequenced region  length (bp) | Amount of CpG sites analysed |
| --- | --- | --- | --- | --- | --- | --- | --- |
| CG6 | PCR→ AGTTAGAGTGAGTGGGTAGTA  PCR← *CAACCCCCCTTCTACACAAAAACT*  Sequencing→ GTGAGTGGGTAGTAAT | 58 | 25 | 86 | chr4:1864164-1864242 | 79 | 3 |
| CG10 | PCR→ TTTAGTGTATTTTTGGGTGTGGTGTTTAT  PCR← *ACTATATACAAATAACAAACCAAACTT*  Sequencing→ TGTTTTGTATAGTAGAGGTTA | 58 | 25 | 186 | chr16:3534508-3534611 | 104 | 1 |
| CG17 | PCR→ GGAGTTTGGGAAGAAAGTTTTT  PCR← *CTACCCATCCTACTACTATCTTCAAAT*  Sequencing→ GAAGAAAGTTTTTGTTGTTAG | 58 | 20 | 167 | chr10:130281707-130281864 | 158 | 3 |
| CG7 | PCR→ AGATTAGGGAAGAGTTATTTGGAAAT  PCR← *CCTAAAACTAAAAAAAACCCATTCTACC*  Sequencing→ TTTTAATTAGTAAGTTATAGGGAG | 58 | 20 | 125 | chr11:110220584-110220663 | 80 | 1 |
| CG14 | PCR→ ATGGTTTATTATTTTTTTATTTGATAATT  PCR← *CACCAATCATTCCTCCAACAAA*  Sequencing→ TTGATAATTTAGTATTAGTTTTAG | 58 | 20 | 98 | chr11:110104045-110104122 | 78 | 2 |
| CG22 | PCR→ TTTTTTTATATGGGGATAGGAATGTGATT  PCR← *AACCCTTCCCCAACCTCTAATACCA*  Sequencing→ GGGGTTTGTTGGTTTT | 58 | 25 | 172 | chr5:1852812-1852926 | 115 | 2 |
| CG23 | PCR→ *GGTAGTGATTATAGTTTGTAGGGGTTTGT*  PCR← AACCCTTCCCCAACCTCTAATACC  Sequencing← ATCTCCACCAAAAAACACCTAA | 60 | 25 | 135 | chr5:1852792-1852864 | 73 | 1 |
| CG16 | PCR→ *AATAGGGTGGTGAAAGGTAGATAAA*  PCR← ACCACAACAACTAAAAACACCTATC  Sequencing← ACTAAAAACACCTATCTCC | 58 | 20 | 131 | chr5:140864546-140864667 | 122 | 11 |
| CG19 | PCR→ GTGAGGGGATTGTTGGAAGAG  PCR← *TTCCCCCACACCTAACACCCATAT*  Sequencing→ AGTAGGAATGTTAATTTGG | 58 | 20 | 183 | chr7:99227410-99227486 | 77 | 4 |
| CG24 | PCR→ *AGAGGAAGTTTTTTTGTTTTATTTTGATA*  PCR← ACATCACTATACTTTCACCCTCT  Sequencing← CTATACTTTCACCCTCTT | 58 | 20 | 123 | chr4:16723341-16723457 | 117 | 6 |
| CG26 | PCR→ AGGTGGTGGTGTTTGTTTT  PCR← *AATACCCACTTAATCCTTAACATCAC*  Sequencing→ TTGTTTTAGGTTGGTTTTT | 58 | 25 | 90 | chr22:44319222-44319299 | 78 | 2 |

**Supplementary table 4. Description of the CpG candidate biomarkers obtained after the epigenome-wide methylation analysis.**

|  | **Methylation**  **EPIC probe ID** | **Genomic location (GRCh37/hg19)** | **Gene symbol** | **Relation to CpG island** | **CpG island** | **Regulatory feature** |
| --- | --- | --- | --- | --- | --- | --- |
| CG1 | cg08942894 | chr15:83563792 | *HOMER2* | Opensea |  | Body |
| CG2 | cg05445162 | chr2:27730181 | *GCKR* | Opensea |  | Body |
| CG3 | cg11113216 | chr12:132847641 | *GALNT9* | Island | chr12:132847640-132847955 | Body |
| CG4 | cg06522913 | chr15:90630711 | *IDH2* | Opensea |  | Body |
| CG5 | cg14838992 | chr14:100814724 | *WARS* | Opensea |  | Body |
| CG6 | cg06148974 | chr4:1864183 |  | Island | chr4:1864182-1864417 |  |
| CG7 | cg12737198 | chr11:110220620 |  | Opensea |  |  |
| CG8 | cg03111938 | chr6:169289230 |  | Island_Shelf | chr6:169286195-169286688 |  |
| CG9 | cg07253636 | chr19:50077766 | *NOSIP* | Opensea |  | 5'UTR |
| CG10 | cg06214087 | chr16:3534568 | *NAA60* | Island_Shore | chr16:3534833-3535066 | Body |
| CG11 | cg25942688 | chr2:27016730 | *CENPA* | Opensea |  | 3'UTR |
| CG12 | cg01987330 | chr3:142797310 |  | Opensea |  |  |
| CG13 | cg22880141 | chr1:28160795 | *SCARNA1 PPP1R8* | Island_Shelf | chr1:28157221-28157788 | TSS200 |
| CG14 | cg10641001 | chr11:110104096 | *RDX* | Opensea |  | Body |
| CG15 | cg01550272 | chr13:115050881 | *UPF3A* | Island_Shelf | chr13:115046754-115048034 | Body |
| CG16^†^ | cg03640756 | chr5:140864593 | *PCDHG* gene cluster | Island | chr5:140864527-140864748 | Body |
| CG17^†^ | cg26024530 | chr10:130281732 |  | Opensea |  |  |
| CG18^†^ | cg22778120 | chr22:45094531 | *PRR5* | Island_Shelf | chr22:45097755-45098801 | 5'UTR |
| CG19^†^ | cg15442105 | chr7:99227437 | *ZNF498* | Opensea |  | Body |
| CG20^†^ | cg04544475 | chr17:56435455 | *RNF43* | Opensea |  | Body |
| CG21^†^ | cg16639692 | chr5:43037666 |  | Island_Shore | chr5:43037259-43037520 |  |
| CG22^†^ | cg14969646 | chr1:3148357 | *PRDM16* | Island_Shore | chr1:3147845-3148081 | Body |
| CG23^†^ | cg04600077 | chr5:1852839 |  | Island_Shore | chr5:1851342-1851564 |  |
| CG24^‡^ | cg14503564 | chr4:16723393 | *LDB2* | Opensea |  | Body |
| CG25^§^ | cg18044585 | chr8:141619425 | *EIF2C2* | Opensea |  | Body |
| CG26^‡^ | cg23653187 | chr22:44319257 | *PNPLA3* | Island_Shore | chr22:44319578-44320513 | TSS1500 |

Regulatory features and relation to CpG island of biomarkers annotated according to the Methylation EPIC Manifest: CpG island: region of at least 200 bp with a CG content > 50% and an observed-to-expected CpG ratio≥0.6; Island-shore: sequences 2 kb flanking the CpG island; Island-shelf: sequences 2 kb flanking shore regions; Opensea: sequences located outside these regions; Body: gene body (intragenic region); TSS200, TSS1500: 200 and 1500 bp upstream the transcription start site, respectively. †Candidate biomarkers derived from the comparison with the external RRBS dataset; ‡candidate biomarkers derived from the NN vs P-AA classification; §candidate biomarker derived from the NN vs D-AA classification.

**Supplementary Figure 1. ROC curve analysis for the 26 candidate biomarkers and *SEPT9* for NN versus AN classification in the biomarker evaluation cohort (n=48).** The red dots indicate the best cut−offs based on the Youden Index method. AN: advanced neoplasia; AUC: area under the curve; NN: no neoplasia; Se: sensitivity; Sp: specificity; YI: Youden Index.


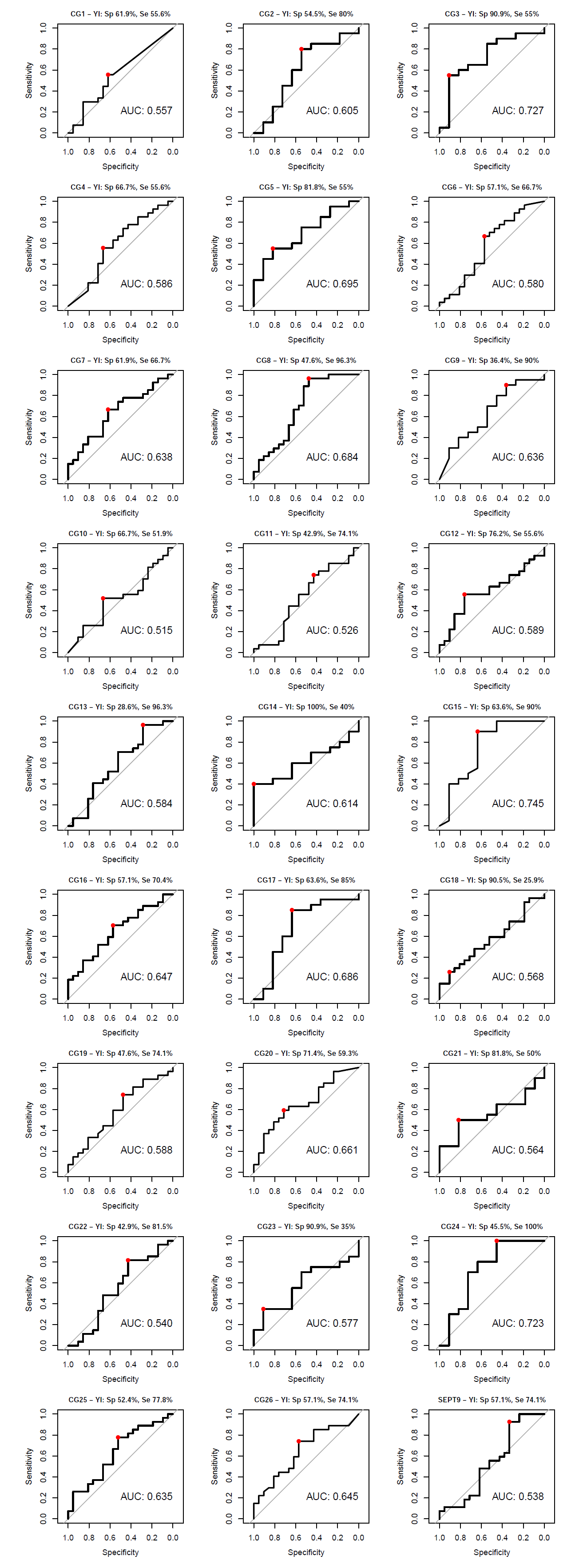


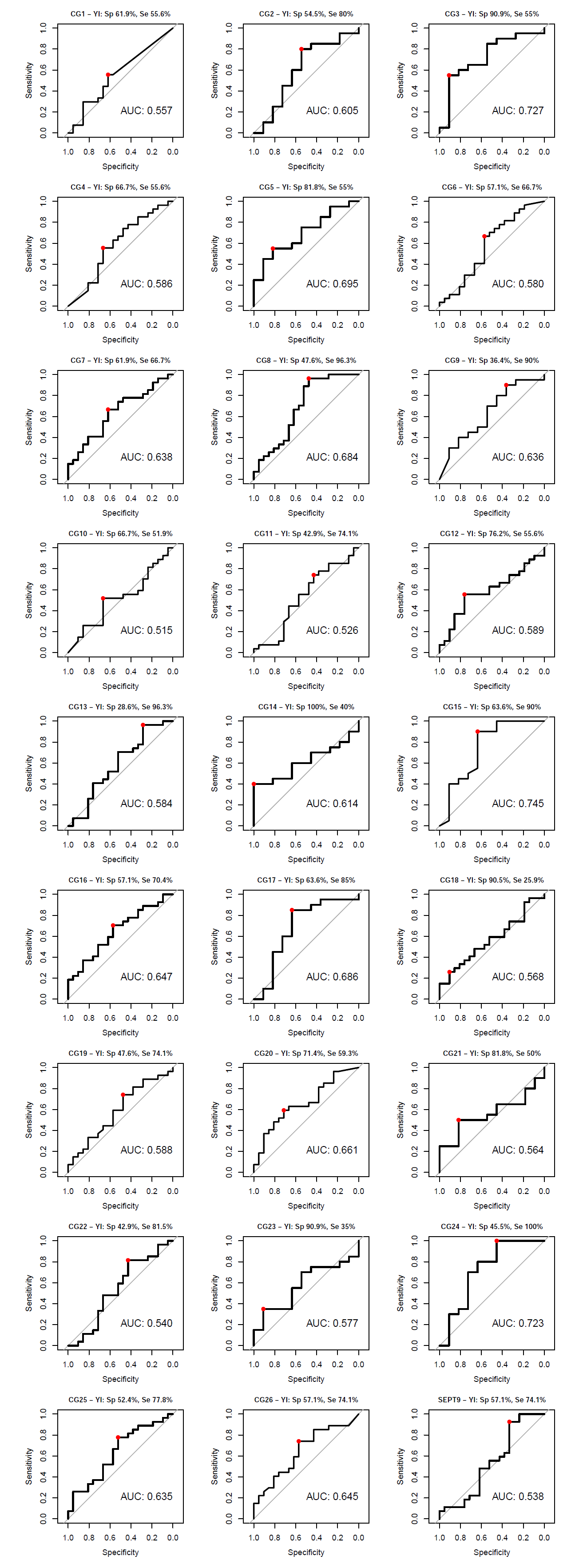
**Supplementary Figure 1 (continuation).**

**Supplementary Figure 2.** **ROC curve analysis for the 20 selected biomarkers and *SEPT9* for NN versus AN classification in the biomarker validation cohort (n=105).** The red dots indicate the best cut−offs based on the Youden Index method. AN: advanced neoplasia; AUC: area under the curve; NN: no neoplasia; Se: sensitivity; Sp: specificity; YI: Youden Index.**
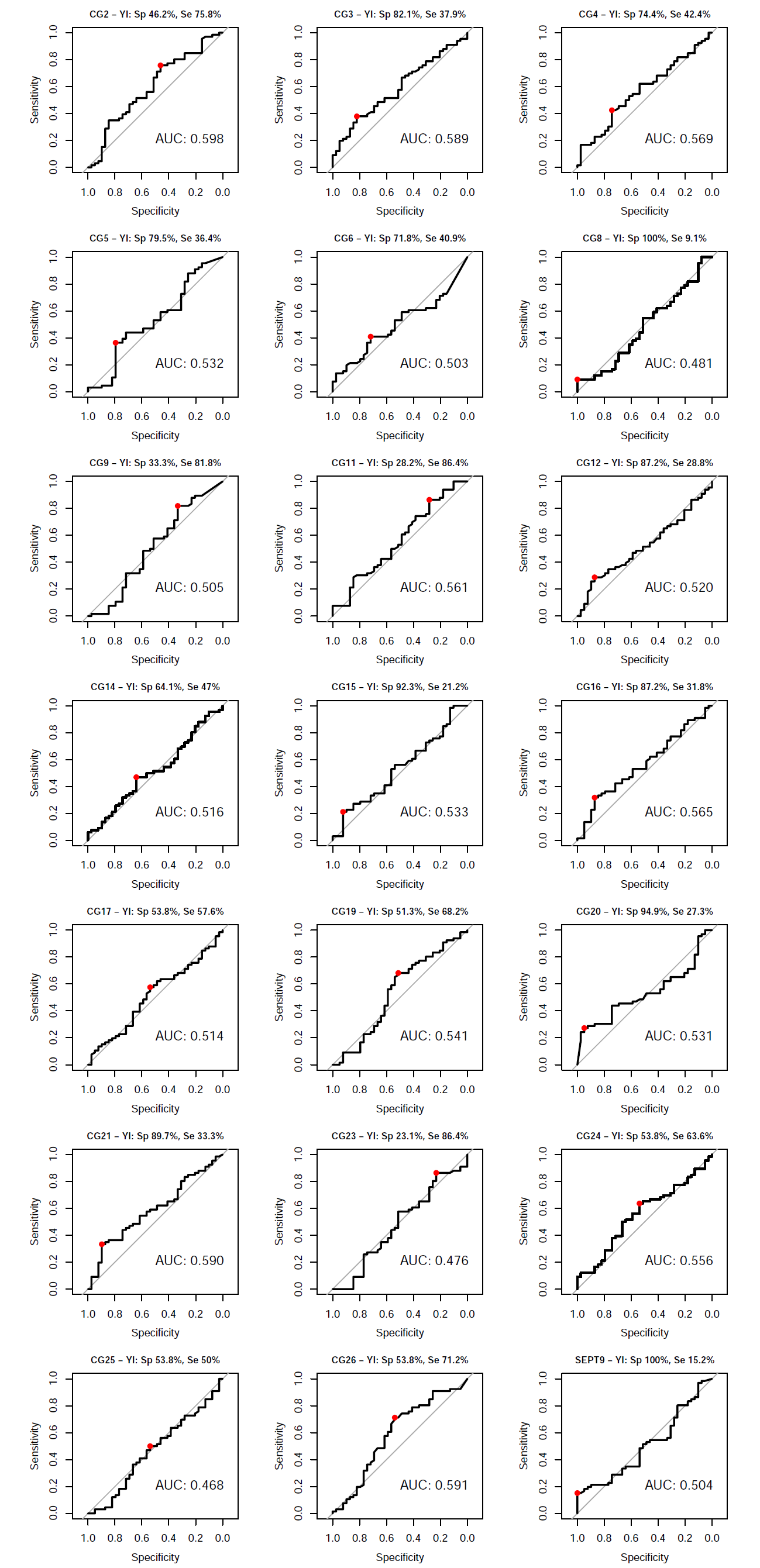
**

**
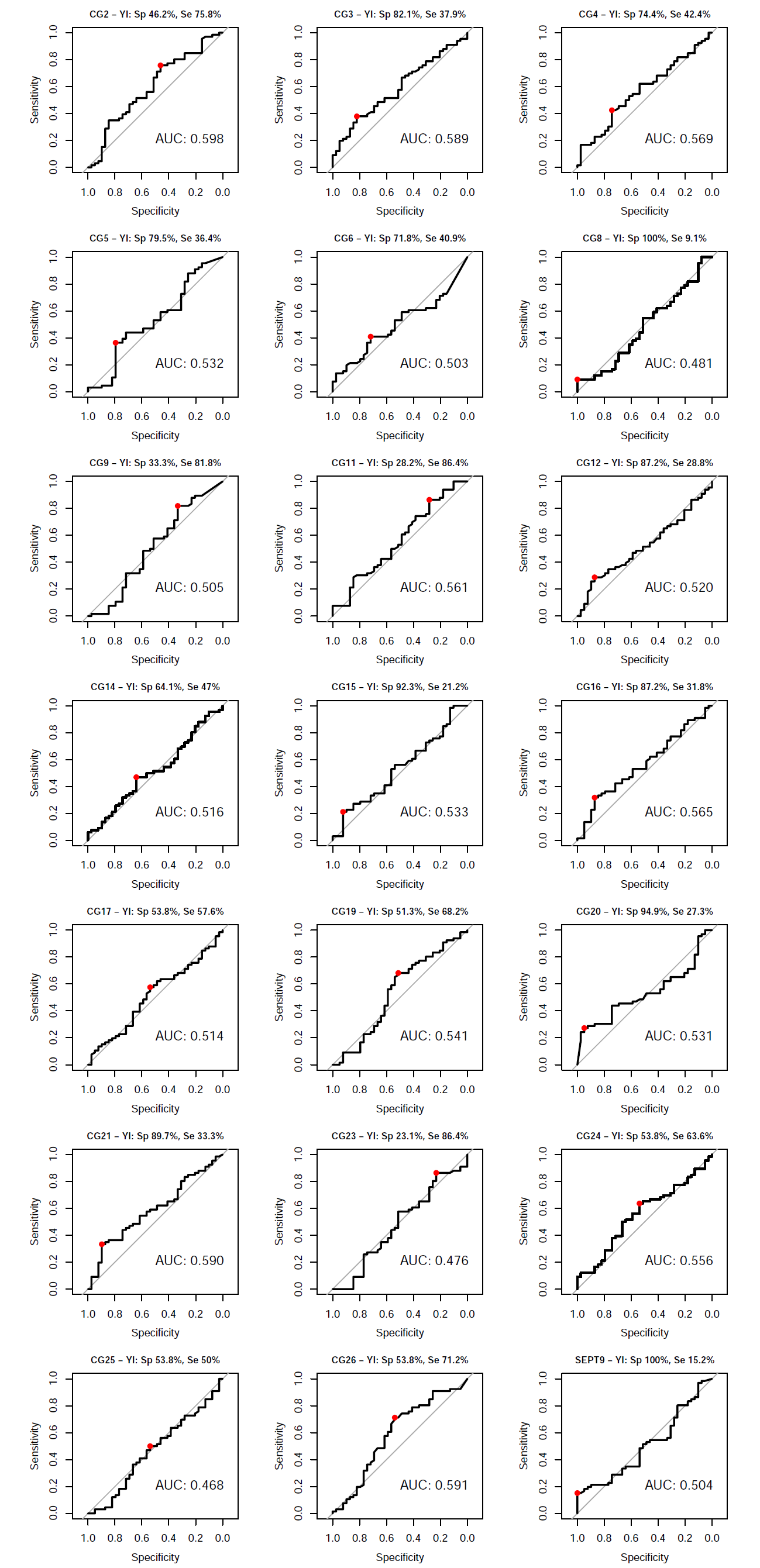
Supplementary Figure 2 (continuation).**

**REFERENCES**

1. R Core Team (2020). R: A language and environment for statistical computing. R Foundation for Statistical Computing. Vienna, Austria;

2. Huber W, Carey VJ, Gentleman R, Anders S, Carlson M, Carvalho BS, et al. Orchestrating high-throughput genomic analysis with Bioconductor. Nat Methods. 2015;12(2):115–21.

3. Aryee MJ, Jaffe AE, Corrada-Bravo H, Ladd-Acosta C, Feinberg AP, Hansen KD, et al. Minfi: A flexible and comprehensive Bioconductor package for the analysis of Infinium DNA methylation microarrays. Bioinformatics. 2014;30(10):1363–9.

4. Pidsley R, Y Wong CC, Volta M, Lunnon K, Mill J, Schalkwyk LC. A data-driven approach to preprocessing Illumina 450K methylation array data. BMC Genomics. 2013;14(1):293.

5. Pidsley R, Zotenko E, Peters TJ, Lawrence MG, Risbridger GP, Molloy P, et al. Critical evaluation of the Illumina MethylationEPIC BeadChip microarray for whole-genome DNA methylation profiling. Genome Biol. 2016;17:208.

6. Hansen KD. IlluminaHumanMethylationEPICanno.ilm10b4.hg19: Annotation for Illumina’s EPIC methylation arrays. 2017.

7. Triche TJ, Weisenberger DJ, Van Den Berg D, Laird PW, Siegmund KD, Siegmund KD. Low-level processing of Illumina Infinium DNA Methylation BeadArrays. Nucleic Acids Res. 2013;41(7):e90.

8. Fortin JP, Triche T, Hansen K. Preprocessing, normalization and integration of the Illumina HumanMethylationEPIC array with minfi. Bioinformatics. 2017;33(4):558–60.

9. Johnson WE, Li C, Rabinovic A. Adjusting batch effects in microarray expression data using empirical Bayes methods. Biostatistics. 2007;8(1):118–27.

10. Leek JT, Johnson WE, Parker HS, Jaffe AE, Storey JD. The sva package for removing batch effects and other unwanted variation in high-throughput experiments. Bioinformatics. 2012;28(6):882–3.

11. Du P, Zhang X, Huang CC, Jafari N, Kibbe WA, Hou L, et al. Comparison of Beta-value and M-value methods for quantifying methylation levels by microarray analysis. BMC Bioinformatics. 2010;11(1):587.

12. Ritchie ME, Phipson B, Wu D, Hu Y, Law CW, Shi W, et al. limma powers differential expression analyses for RNA-sequencing and microarray studies. Nucleic Acids Res. 2015;43(7):e47.

13. Mansell G, Gorrie-Stone TJ, Bao Y, Kumari M, Schalkwyk LS, Mill J, et al. Guidance for DNA methylation studies: Statistical insights from the Illumina EPIC array. BMC Genomics. 2019;20(1):1–15.

14. Peña EA, Slate EH. Global validation of linear model assumptions. J Am Stat Assoc. 2006;101(473):341–54.

15. Lagani V, Athineou G, Farcomeni A, Tsagris M, Tsamardinos I. Feature Selection with the R Package MXM : Discovering Statistically Equivalent Feature Subsets . J Stat Softw. 2017;80(7).

16. Meyer D, Dimitriadou E, Hornik K, Weingessel A, Leisch F. e1071: Misc Functions of the Department of Statistics, Probability Theory Group (Formerly: E1071). TU Wien; 2020.

17. Liaw A, Wiener M. Classification and Regression by randomForest. R News. 2002;2(3):18–22.
